# Positron emission tomography and functional characterization of a complete PBR/TSPO knockout

**DOI:** 10.1038/ncomms6452

**Published:** 2014-11-19

**Authors:** Richard B. Banati, Ryan J. Middleton, Ronald Chan, Claire R. Hatty, Winnie Wai-Ying Kam, Candice Quin, Manuel B. Graeber, Arvind Parmar, David Zahra, Paul Callaghan, Sandra Fok, Nicholas R. Howell, Marie Gregoire, Alexander Szabo, Tien Pham, Emma Davis, Guo-Jun Liu

**Affiliations:** 1 Life Sciences, Australian Nuclear Science and Technology Organisation, Locked Bag 2001, Kirrawee DC, New South Wales 2232, Australia; 2 Brain & Mind Research Institute, The University of Sydney, Sydney, New South Wales 2006, Australia; 3 Medical Imaging & Radiation Sciences, Faculty of Health Science and Brain & Mind Research Institute, The University of Sydney, Sydney, New South Wales 2006, Australia; 4 National Imaging Facility, Sydney, Camperdown, New South Wales 2006, Australia; 5 Sydney Medical School, The University of Sydney, Sydney, New South Wales 2006, Australia; 6 Centre for Translational Neuroscience, University of Wollongong, Wollongong, New South Wales 2522, Australia

## Abstract

The evolutionarily conserved peripheral benzodiazepine receptor (PBR), or 18-kDa translocator protein (TSPO), is thought to be essential for cholesterol transport and steroidogenesis, and thus life. TSPO has been proposed as a biomarker of neuroinflammation and a new drug target in neurological diseases ranging from Alzheimer’s disease to anxiety. Here we show that global C57BL/6-*Tspo*^*tm1GuWu(GuwiyangWurra)*^-knockout mice are viable with normal growth, lifespan, cholesterol transport, blood pregnenolone concentration, protoporphyrin IX metabolism, fertility and behaviour. However, while the activation of microglia after neuronal injury appears to be unimpaired, microglia from ^*GuwiyangWurra*^TSPO knockouts produce significantly less ATP, suggesting reduced metabolic activity. Using the isoquinoline PK11195, the ligand originally used for the pharmacological and structural characterization of the PBR/TSPO, and the imidazopyridines CLINDE and PBR111, we demonstrate the utility of ^*GuwiyangWurra*^TSPO knockouts to provide robust data on drug specificity and selectivity, both *in vitro* and *in vivo*, as well as the mechanism of action of putative TSPO-targeting drugs.

## Abstract (web summary)

The 18-kDa translocator protein (TSPO) has been implicated in steroid biogenesis and neuroinflammation. Here, the authors create viable and fertile global TSPO knockout mice, challenging the assumption that TSPO is essential for mouse development but suggesting that it may have a role under certain disease conditions.

## Graphical abstract

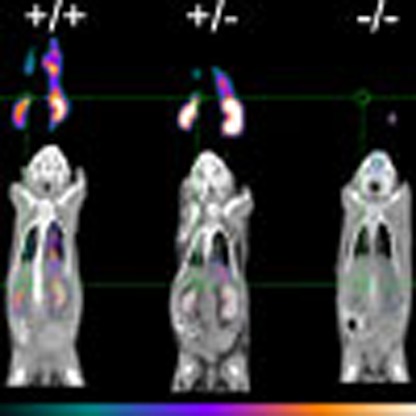

According to current concepts, the most important function of the mitochondrial membrane protein peripheral benzodiazepine receptor (PBR)/18-kDa translocator protein (TSPO) is the regulation of steroid hormone production by helping to translocate cholesterol, the precursor to pregnenolone, across the aqueous mitochondrial intermembrane space, a view emphasized by the recent renaming of the PBR as translocator protein (TSPO)^1^,^2^. A large body of literature^2^,^3^,^4^ on the regulatory influence of the PBR/TSPO on steroid-mediated homeostasis, including the reported embryonic lethal effects of a *Tspo* knockout^5^, has established the PBR/TSPO as essential for life. Its critical role as an endocrine regulator is the prevailing explanation for the observed actions of PBR/TSPO binding drugs across an exceptionally broad therapeutic spectrum from the anti-inflammatory treatment of Alzheimer’s disease^6^ to anxiolysis without direct effects on the central GABA_A_ receptor protein complex^4^,^7^. However, recent observations that a conditional knockout in testicular Leydig cells appeared not to affect hormone production^8^ have controversially been interpreted as evidence that the PBR/TSPO, unlike the steroidogenic acute regulatory protein (StAR)^9^,^10^, is not an essential requirement for steroid hormone biosynthesis^11^,^12^. No further data indicating other potential impairments have been reported.

An important observation that has underpinned the growing interest related to the PBR/TSPO is the regularly seen increase of the PBR/TSPO in areas of brain injury and during ‘neuroinflammation’, most prominently in activated microglia^1^,^13^,^14^.

Our study provides a first extensive reference description of the constitutive phenotype of a global *Tspo* knockout animal model *in vitro* and *in vivo*. It addresses some of the fundamental questions, in regard to the long-term viability of animals without any potentially compensating PBR/TSPO-expressing cells, whether they have altered steroid levels or any other loss of function and whether specificity and selectivity can be assumed for some of the most widely used PBR/TSPO-ligands.

## Results

### Confirmation of global Tspo^+/−^ and Tspo^*−/−*^ knockout

**Figure 1:**
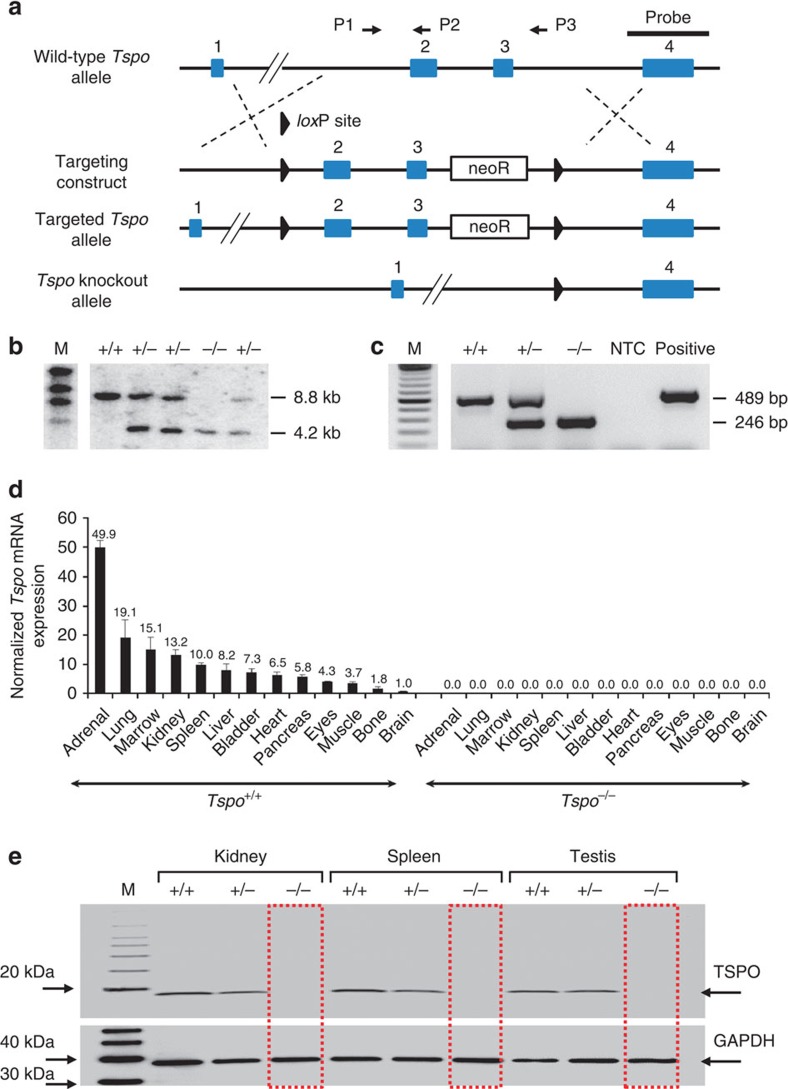
Generation and confirmation of global *Tspo*^*−/−*^ mice. (**a**) The *Tspo* gene was knocked out using a targeting construct with *lox*P sites flanking exons 2 and 3 (also see Supplementary Fig. 1). (**b**,**c**) Southern blot analysis (**b**) and PCR (BV2 mouse microglia were used as the positive control) (**c**) demonstrated the correct targeting of the *Tspo* gene. (**d**,**e**) *Tspo* mRNA expression across 13 tissues (triplicates, mean and standard deviation; normalized to *Gapdh* and *Actb*) (**d**) and measurement of TSPO protein by western blot of lysates from the kidney, spleen and testis (**e**) confirmed the complete absence of any *Tspo* gene product in *Tspo*^*−/−*^ mice.

Removal of exons 2 and 3 of the *Tspo* gene resulted in viable *Tspo*^+/−^ and *Tspo*^*−/−*^ animals. Following the removal of exons 2 and 3 only exons 1 and 4 remain, both of which do not contain any start codons in the TSPO reading frame. Therefore, no TSPO protein, or truncated TSPO protein can be produced (Fig. 1a). A more detailed illustration of how the loss of exons 2 and 3 and subsequent merger of exon 1 and exon 4 cannot give rise to any functional fragment of the PBR/TSPO but possibly only an unrelated protein with no sequence similarity is shown in Supplementary Fig. 1.

**Figure 2:**
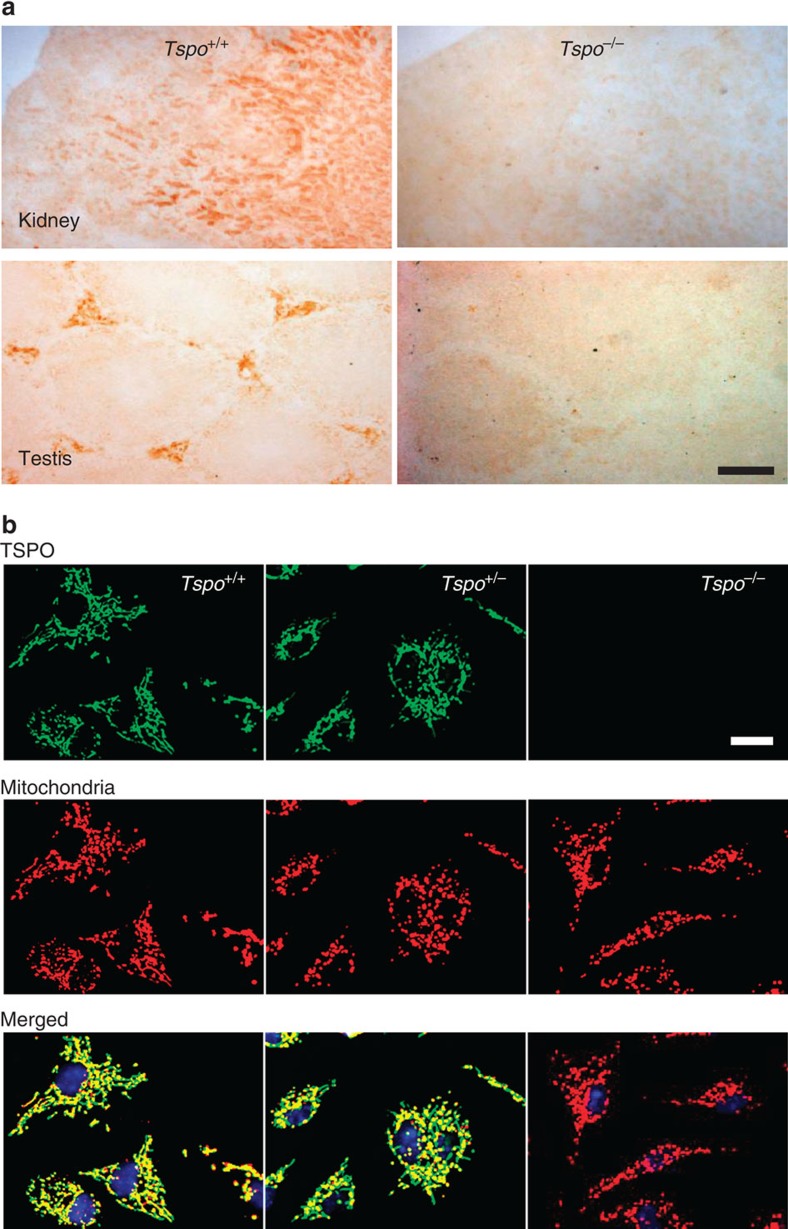
Confirmation of global *Tspo* knockout mice with immunostaining. (**a**) Anti-TSPO antibody staining showed the presence of TSPO (here shown in the kidney and testis) in the wild-type and absence in the knockout *Tspo*^*−/−*^ mice. The slides were the same as previously used for autoradiography with the selective TSPO-binding ligand [^3^H]PK11195 (Fig. 4). (**b**) Antibody staining of *Tspo*^+/+^ and *Tspo*^+/−^ macrophages validates mitochondria as the primary site of the TSPO, which is entirely absent in *Tspo*^*−/−*^ mice. No obvious difference in intracellular density or distribution of the mitochondria was detected in the *Tspo*^*−/−*^ mice (green=TSPO; red=mitochondria; yellow=merged image; blue=nucleus; scale bars: (**a**) 500 μm and (**b**) 20 μm).

**Figure 3:**
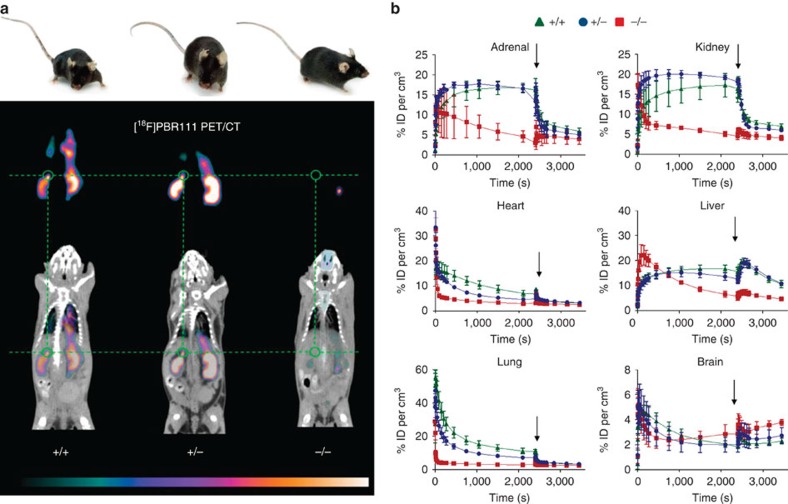
No constitutive TSPO ligand binding in *Tspo*^*−/−*^ mice. (**a**) *Tspo*^+/+^, *Tspo*^+/−^ and *Tspo*^*−/−*^ mice are identical in external appearance and general behaviour. However, *in vivo* imaging (8 males of the same age for each genotype) with PET/CT using the radioligand [^18^F]PBR111, the ^18^F-labelled analogue to [^125^I]CLINDE, strikingly illustrates that (apart from occasional signals originating from the excretory pathways, such as gut and urinary bladder) *Tspo*^*−/−*^ mice show no ligand binding (thus also demonstrating the selectivity of the used ligand), while both *Tspo*^+/+^ and *Tspo*^+/−^ mice have the characteristic distribution of ligand binding, that is, in organs with known high TSPO expression, notably kidney and adrenal gland (green circle). The images are displayed with the colour scaling and are directly comparable (highest values are white). The time-point frame of the PET images is 15–20 min after injection and scaling is 3.8–17.9% ID per cm^3^. (**b**) The kinetics of [^18^F]PBR111, and the displacement of the radioligand after injection (indicated by arrows) with cold PBR111 (1 mM) to establish non-specific binding, demonstrates that *Tspo*^*−/−*^ mice do not have specific binding of [^18^F]PBR111 in any organs, while the ligand kinetic in *Tspo*^+/+^ and *Tspo*^*+/−*^ mice indicates specific binding (ID= injected dose; *n*=4 for each genotype; error bars denote standard deviation).

**Figure 4:**
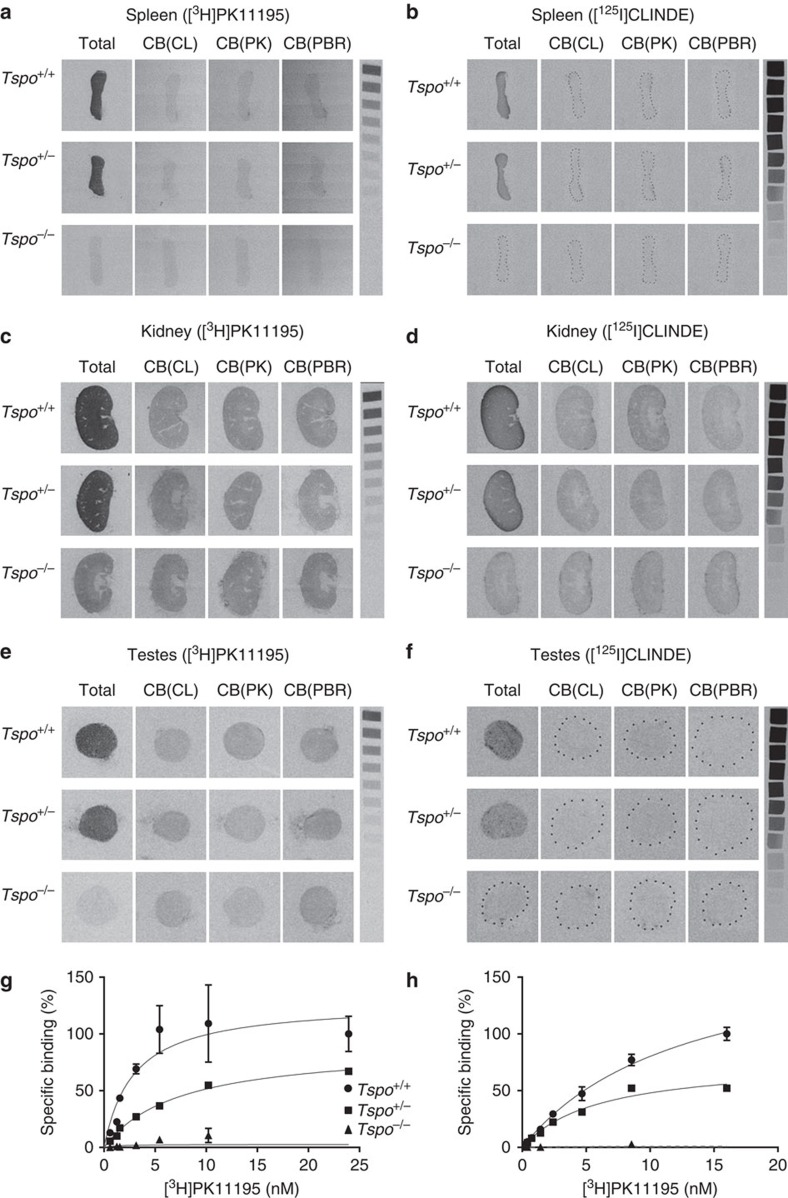
Comparative receptor autoradiography and membrane binding. (**a**–**f**) The receptor autoradiographs (16 μm sections) show total binding of [^3^H]PK11195 (1 nM) and [^125^I]CLINDE (3 nM) as well as competitive binding with 10 μM unlabelled CLINDE (CB(CL)), PK11195 (CB(PK)) and PBR111 (CB(PBR)) in the spleens (**a**,**b**), kidneys (**c**,**d**) and testes (**e**,**f**) of *Tspo*^*+/+*^, *Tspo*^*+/−*^ and *Tspo*^*−/−*^ mice. Specific binding of 3 nM [^125^I]CLINDE and 1 nM [^3^H]PK11195 is clearly visible in tissue sections from *Tspo*^*+/+*^ and *Tspo*^+/−^ mice and is displaceable by the unlabelled ligands. There is no specific binding in *Tspo*^*−/−*^ tissue. (**g**,**h**) Specific binding using [^3^H]PK11195 in testicular tissue (**g**) and kidney tissue (**h**) (*n*=3 for each genotype *Tspo*^+/+^, *Tspo*^+/−^ and *Tspo*^*−/−*^). *Tspo*^+/−^ mice (kidney (Bmax and Kd): 45,361.0 fmol per mg and 5.94 nM; testes: 3,696.0 fmol per mg and 7.32 nM) have approximately half the binding of *Tspo*^*+/+*^mice (kidney: 108,934.0 fmol per mg and 12.90 nM; testes: 4,920.0 fmol per mg and 2.66 nM) while *Tspo*^*−/−*^mice have no appreciable binding. Curves represent non-linear regression of experimentally obtained data points and data are expressed as percentage relative to *Tspo*^*+/+*^ specific binding. Error bars denote standard deviation.

**Figure 5:**
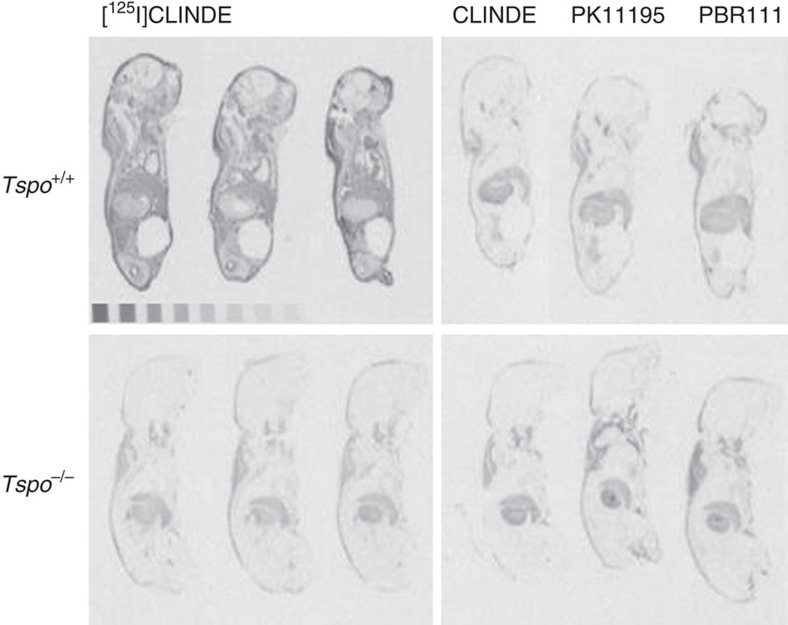
Whole-body receptor autoradiography of neonatal mice. Receptor autoradiography using the TSPO ligand [^125^I]CLINDE on whole bodies of 2-day-old neonatal mice. The autoradiographs show total binding of [^125^I]CLINDE (3 nM) as well as competitive displacement binding with 10 μM unlabelled CLINDE (CB(CL)), PK11195 (CB(PK)) and PBR111 (CB(PBR)). Specific binding of [^125^I]CLINDE is clearly visible in the *Tspo*^+/+^ mice and is displaceable by all three unlabelled ligands. Non-specific binding is seen in areas of nuchal fat or stomach content.

**Figure 6:**
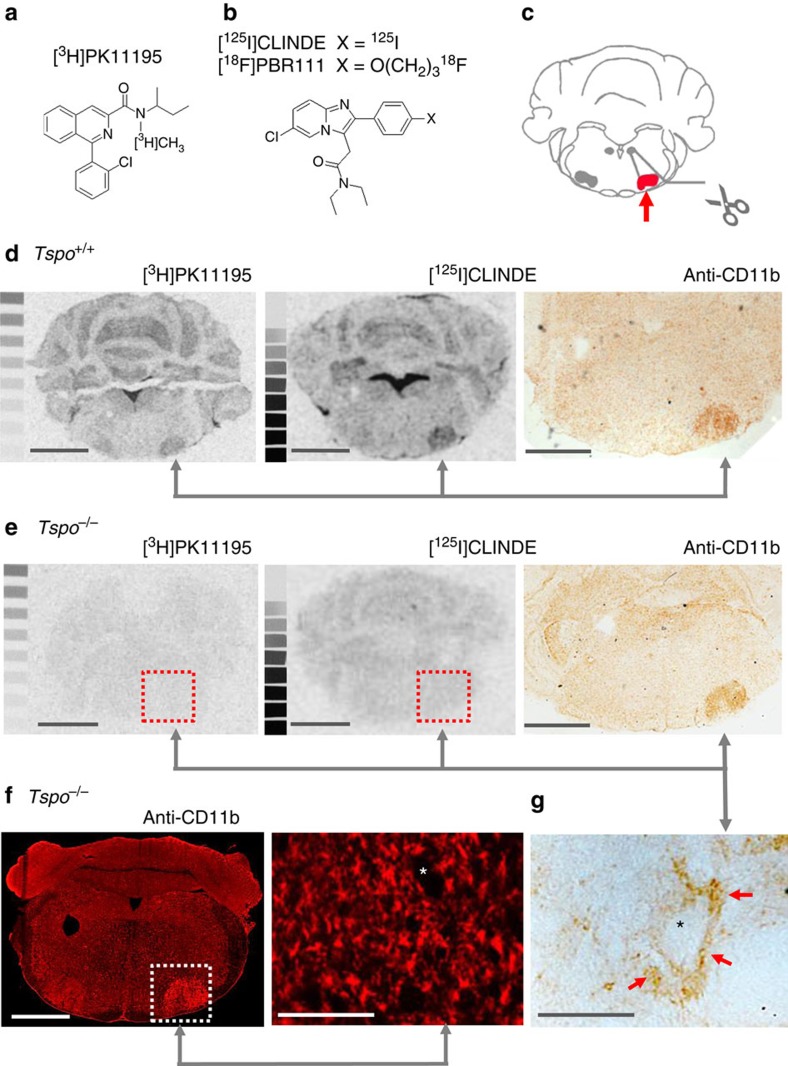
No inducible TSPO ligand binding in *Tspo*^*−/−*^ mice. (**a**,**b**) TSPO-binding ligands PK11195 and CLINDE/PBR111. (**c**) Axotomy of the facial nerve induces a retrograde neuronal reaction and highly reproducible microglial activation in the injured facial nucleus. (**d**) Autoradiography with [^3^H]PK11195 and [^125^I]CLINDE and immunohistochemical staining of the microglial activation marker CD11b on consecutive brain sections confirmed the previously reported localized induction of TSPO ligand binding in the injured facial nucleus contemporaneous to the activation of microglia of *Tspo*^*+/+*^ animals. (**e**) In contrast, no binding of [^3^H]PK11195 and [^125^I]CLINDE could be induced in *Tspo*^*−/−*^ mice despite the undiminished presence of activated microglia in the injured facial nucleus, thus providing evidence that the high selectivity of [^3^H]PK11195 and [^125^I]CLINDE for their respective binding sites on the TSPO is retained in pathologically changed tissue. (**f**) Immunofluorescent anti-CD11b staining of activated microglia in the injured facial nucleus of *Tspo*^*−/−*^ mice revealed no obvious differences in microglial activation, with its characteristic localization of activated, perineuronal microglia, here (**g**) shown as higher magnification of the above section. Scale bars in (**d**,**e**) and the left image of (**f**) denote 1 mm, and that in the right image of (**f**) and in (**g**) 100 μm. The asterixes in (**g** and **f**) indicates the soma of neurons.

The targeted deletion of *Tspo* and complete loss of TSPO protein was confirmed by Southern blot, PCR, RT-PCR, RT-qPCR, Western blot, (Fig. 1b–e and Supplementary Fig. 1), specific antibody staining against amino acids 156–169 at the C-terminus of the PBR/TSPO in tissues and macrophages from *Tspo*^+/+^, *Tspo*^+/−^ and *Tspo*^*−/−*^ mice (Fig. 2), *in vivo* tracer kinetic PET/CT studies using the PBR/TSPO ligand [^18^F]PBR111 (Fig. 3), receptor-autoradiography and membrane receptor binding (Figs 4 and 5) using [^3^H]PK11195 (Fig. 6a) and [^125^I]CLINDE (Fig. 6b).

In addition, PBR/TSPO receptor membrane-binding data as well as extensive receptor autoradiographic validation for all major organs and the whole body of neonatal mice in all three genotypes confirm the absence of the PBR/TSPO protein in the *Tspo*^*−/−*^ mice and the high selectivity of [^3^H]PK11195 in tissues where the PBR/TSPO is present (Fig. 4a,c,e,g,h and Supplementary Fig. 2). Further, we demonstrate *in vivo* and *in vitro* the high selectivity of [^18^F]PBR111 and [^125^I]CLINDE (Figs 3, 4b,d,f and 5), which are thus the first new compounds for the PBR/TSPO validated in animals with a null background of any constitutive, or lesion-induced, specific TSPO binding.

Importantly, we show that in *Tspo*^*−/−*^ animals, unlike in the normal wild-type, the microglial cell response in the facial nucleus after peripheral facial nerve lesion is not associated with an increase in the binding of the PBR/TSPO ligands, [^3^H]PK11195 and [^125^I]CLINDE. This demonstrates that in pathologic tissue changes the selectivity of [^3^H]PK11195 and [^125^I]CLINDE holds true and no additional non-selective binding emerges (Fig. 6a–e). Our data also indicate that the early stage of perineuronal microglial activation, with its typical change in microglial morphology, is not noticeably influenced by the loss of the PBR/TSPO and that the neuro-glial signaling mechanism remains intact (Fig. 6f,g).

**Figure 7:**
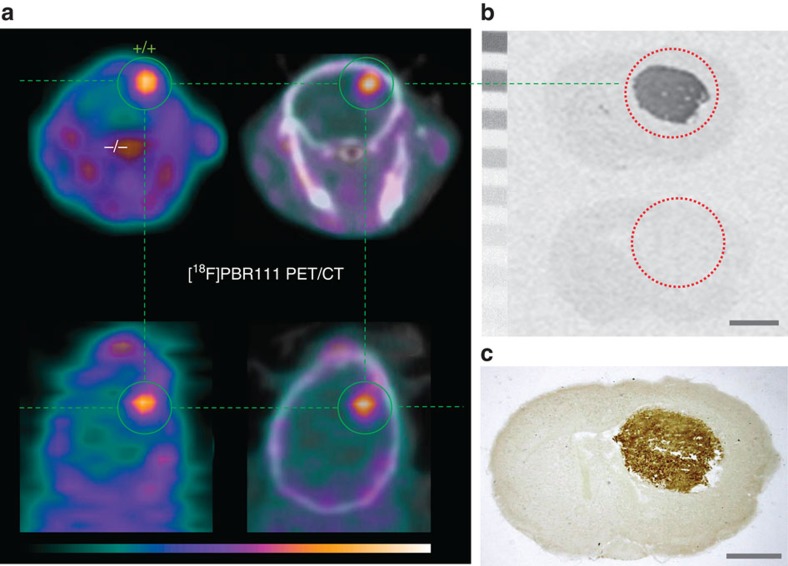
PBR/TSPO-expressing brain tumour in global *Tspo*^*−/−*^ mouse brain. (**a**) [^18^F]PBR111 PET/CT demonstrates a high-contrast signal confined exclusively to the *Tspo*^+/+^ brain tumour, while *Tspo*^*−/−*^ surrounding brain shows no ligand binding. (**b**,**c**) For the same animal shown in (**a**), *in vitro* autoradiography with [^3^H]PK11195 (**b**) and subsequent immunohistochemical staining with anti-TSPO antibody on the same brain sections (**c**) confirm that PBR/TSPO ligand binding is strictly limited to the brain tumour region and no PBR/TSPO binding or recognition sites are present in *Tspo*^*−/−*^ tissue. Scale bars, 1 mm. The PET images show the time frame from 5 to 10 min after injection and scaling is 0–5.3% ID per cm^3^ for (**a**).

We further demonstrate the background-free detection of syngeneic PBR/TSPO-expressing glioma cells growing in the brains of *Tspo*^*−/−*^ animals *in vitro* and *in vivo* using the selective PBR/TSPO ligands, [^3^H]PK11195 and [^18^F]PBR111, as well as antibody staining against the PBR/TSPO. This approach tests simultaneously for the absence (respectively presence) of several recognition or binding domains that make up the full PBR/TSPO, whereby the PBR/TSPO-expressing tumour serves as an internal positive control within the same animal. As predicted from the readable sequences remaining after the deletion of exons 2 and 3, the tissue of *Tspo*^*−/−*^ animals cannot express any functional domains of the PBR/TSPO or similar proteins, whereas the *Tspo*^+/+^ tumours express the protein as expected (Fig. 7 and Supplementary Fig. 3).

### General health and behavioural phenotyping

**Figure 8:**
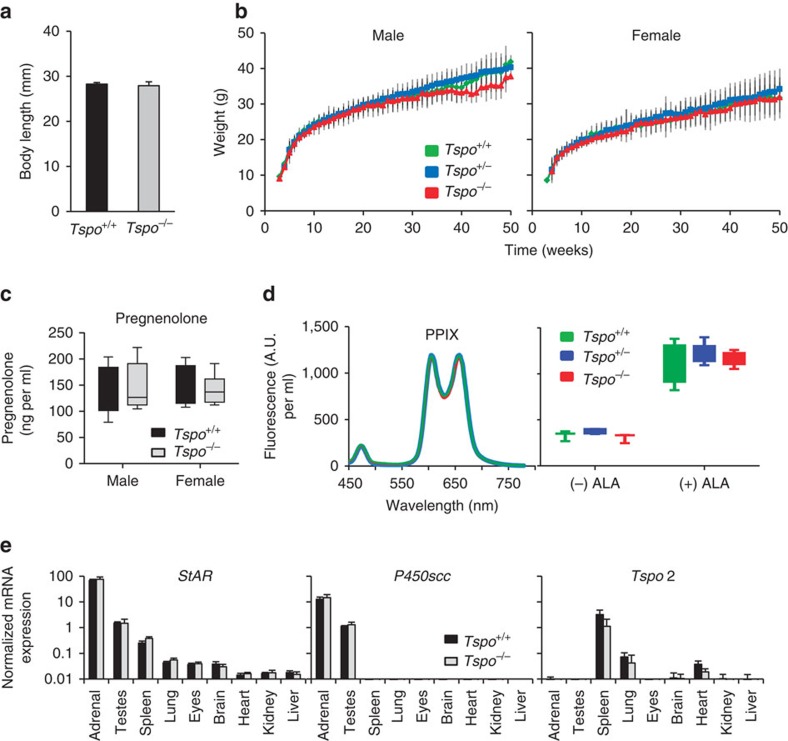
Functional characterization of global *Tspo*^*−/−*^ mice. (**a**) No significant differences in body length between *Tspo*^+/+^ (*n*=5) and *Tspo*^*−/−*^ (*n*=9) neonatal 1-day-old mice are apparent (Student’s *t*-test). (**b**) Likewise, there is no significant difference (ANOVA) in the weight gain trajectory measured from 4 to 83 weeks in *Tspo*^*+/+*^, *Tspo*^+/−^ and *Tspo*^*−/−*^ mice within the same sex (*n*=10–80 animals per time point for weeks 4–43 per group; *n*=3–44 per time point for weeks 44–83). Independent from genotypes, females weigh significantly less than males, as is known to be the case for this mouse strain (**b**). (**c**–**e**) No significant differences between *Tspo*^*+/+*^ and *Tspo*^*−/−*^ mice are found in blood pregnenolone concentrations (*n*=4 for all groups; Student’s *t*-test) (**c**); blood protoporphyrin IX (PPIX; left: three typical closely overlapping spectra from the different genotypes after addition of 5-aminolevulinic acid (ALA), right: no significant differences in levels of PPIX (PPIX per ml of blood in arbitrary units (A.U.); *n*=3–5 per genotype; Student’s *t*-test)) (**d**); and mRNA expression of *steroidogenic acute regulatory* (*StAR*) protein, *P450scc* and *Tspo2* in nine organs (normalized to reference genes *Actb* and *Gapdh*, *n*=3 per genotype) (**e**). Error bars denote standard deviation.

The observation of over 600 animals did not reveal any overt clinical impairment under standard diet (Supplementary Table 1) and normal housing conditions, nor increase in incidental pathologies in either heterozygous *Tspo*^+/−^ or homozygous *Tspo*^*−/−*^ animals, both having the same litter size, length of 1-day-old pups, sex ratio, and subsequent growth rate and weight increase from 3 to 50 weeks (weekly measurement) as the colony control wild-type *Tspo*^+/+^ animals (Fig. 8a,b). Follow-up monthly measurements from 50 to 80 weeks failed to reveal any significant genotype-dependent weight differences in older animals.

Likewise, there was no indication of a decrease in fertility or lifespan, the oldest animals having so far exceeded 24 months without illness. Tests of sperm viability and function yielded no differences between sperm from *Tspo*^+/+^ (mean motility: 82.7±9.33%; mean progress: 47.7±10.2%; mean velocities average path velocity (VAP): 98.6±13.0 μm s^−1^, straight line velocity (VSL): 65.7±8.90 μm s^−1^ and curvilinear velocity (VCL): 179±20.2 μm s^−1^) and *Tspo*^*−/−*^ animals (mean motility: 86.1±5.74%; mean progress: 49.6±5.32%; mean velocities VAP: 105±6.54 μm s^−1^, VSL: 67.5±5.08 μm s^−1^ and VCL: 197±11.2 μm s^−1^). Standard open field, emergence, light/dark preference and elevated plus maze tests revealed similar behaviour across all genotypes and both sexes (Supplementary Table 2).

### Cholesterol and pregnenolone biosynthesis

In both, male and female *Tspo*^*−/−*^ mice, all serum pregnenolone concentrations, as measured by enzyme-linked immunosorbent assay (ELISA), were within the normal range seen in the colony control wild-type (*Tspo*^+/+^: males 131±46.5 ng ml^−1^, females 150±36.5 ng ml^−1^; *Tspo*^*−/−*^: males 145±44.8 ng ml^−1^, females 141±26.8 ng ml^−1^) (Fig. 8c).

The enzymatic conversion of ^3^H-cholesterol to pregnenolone by P450scc over a period of up to 2 h as a read-out of mitochondrial cholesterol transport revealed no statistically significant differences in mean percentage conversion rates between all genotypes (*Tspo*^+/+^: 14.35±4.6% (std); *Tspo*^+/−^: 14.28±3.6% (std); *Tspo*^*−/−*^: 15.6±4.0% (std)).

### Haematological analysis and blood biochemistry

Analysis of blood, including cell sorting, demonstrated that the blood phenotype in both sexes remained largely unaffected by the heterozygous or homozygous loss of the *Tspo* gene, the only exception being a statistically significant trend increase in natural killer (NK) cells in female *Tspo*^*−/−*^ mice compared to female *Tspo*^+/+^ (Supplementary Table 3). Thus, the *Tspo*^*−/−*^ mice did not reveal the changes in cellular blood composition seen in zebrafish *tspo* gene silencing experiments^15^.

The concentrations of protoporphyrin IX (PPIX), thought to be an endogenous PBR/TSPO ligand, in the blood of *Tspo*^+/+^, *Tspo*^+/−^ and *Tspo*^*−/−*^ mice were indistinguishable and increased similarly after treatment with 5-aminolevulinic acid (ALA), the first intermediate in haem biosynthesis and a precursor of PPIX (Fig. 8d), thus indicating normal haem metabolism.

### Absence of compensatory transcriptomic regulation

Likewise, quantitative RT-PCR showed that the gene expression profiles for *StAR*, *P450scc* and *Tspo*2 across the major organs known to express the *Tspo* were similar across genotypes, indicating that the global loss of *Tspo* had not led to any compensatory transcriptomic regulation of those genes thought to be most closely linked to PBR/TSPO as a regulator of steroidogenesis (Fig. 8e).

### Mitochondrial respiration and ATP production in microglia

**Figure 9:**
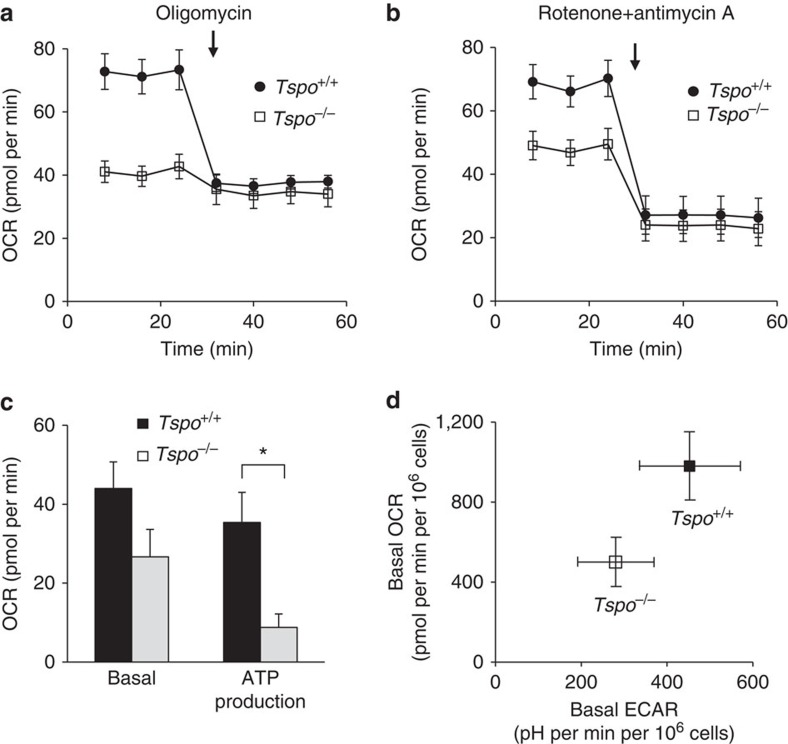
Decreased metabolic activity in *Tspo*^*−/−*^ microglia. (**a**–**c**) The basal, that is, mitochondrial and non-mitochondrial, oxygen consumption rate (OCR) is consistently and significantly lower in *Tspo*^*−/−*^ microglia compared to *Tspo*^*+/+*^ microglia (*Tspo*^*−/−*^: 49.0±16.3 pmol per min; *n*=95; *Tspo*^*+/+*^: 81.0±21.8 pmol per min, *n*=154; Student’s *t*-test), while OCR after application of the ATPase inhibitor oligomycin (3 μM) is reduced to similar levels in both genotypes (*n*=11–14 per group) (**a**). Thus, the oligomycin-inhibitable mitochondrial ATP production in *Tspo*^*−/−*^ microglia is significantly lower than in *Tspo*^*+/+*^ microglia (*P≪0.01; Student’s *t*-test) (**c**). Basal mitochondrial OCRs in *Tspo*^*−/−*^ and *Tspo*^*+/+*^ microglia after inhibition of the electron transport chain complexes I and III with 10 μM rotenone+10 μM antimycin A are reduced by similar amounts (**c**) and to similar levels (**b**) (*n*=11–14 per group). (**d**) Metabolic activity is decreased in *Tspo*^*−/−*^ microglia. Basal OCR vs ECAR (extracellular acidification rate) shows that both OCR and ECAR in *Tspo*^*−/−*^ microglia (*n*=95) are lower than those in *Tspo*^*+/+*^ microglia (*n*=154). Error bars denote standard deviation for (**a**–**d**). Further extensive hematological and behavioural data are presented in Supplementary Tables 2 and 3.

Measurements of mitochondrial respiration and ATP production in primary cultures of mitochondria-rich microglia from *Tspo*^+/+^ and *Tspo*^*−/−*^ mice using the Seahorse XF96 Cell Analyzer and selective inhibitors of the electron transport chain revealed that *Tspo*^*−/−*^ mitochondria, compared to *Tspo*^+/+^, have a consistently and significantly reduced ATP production as determined by inhibition of complex V (ATP synthase) with oligomycin (Fig. 9).

## Discussion

Orthologues of the PBR, or TSPO, are found widely among eubacteria, archaea and eukaryotes^2^,^3^. Most members of the PBR/TSPO protein family contain a specific binding site for the isoquinoline carboxamide PK11195, which has been used to pharmacologically define the PBR/TSPO and distinguish it from other benzodiazepine-binding receptors, such as the GABA_A_ receptor protein complex^1^,^3^,^16^. An evolutionarily younger C-terminal cholesterol recognition amino-acid consensus (CRAC) domain is primarily found in the animal phylum. A paralogue of the PBR/TSPO, the TSPO2, exists in birds and mammals as the result of a gene duplication, which has retained the CRAC domain but lost the isoquinoline-binding site^17^.

In light of the evolutionary pressure to conserve the gene, its documented influence on steroidogenesis and the previously observed embryonic lethality caused by the knockout of either the *Tspo*^5^ or the endogenous PBR/TSPO ligand, Acyl-CoA binding protein^18^, the survival and overtly normal phenotype of a global *Tspo* knockout animal might seem unexpected. However, the evolutionary preservation of an ancient gene does not necessarily imply that it is essential, that is, embryonic lethal, nor that an ancient and non-essential gene is less likely to be relevant for the emergence of disease. On the contrary, phylostratigraphic analysis of the human genome suggests that non-essential ancient genes predominate among the disease-associated genes^19^. PBR/TSPO may be considered a non-essential disease gene that lies at the functional periphery of the interactome with more limited direct interactions than an *in utero* essential hub gene^20^. Consequently, the functional impact on the *Tspo* knockout would be predicted to be discreet.

Our observation that microglia isolated from *Tspo* knockouts have altered oxygen consumption and ATP synthesis rates points to a potential latent phenotype that may be elicited under those disease conditions where a significant regulation of the PBR/TSPO is known to occur^13^,^14^. This raises the intriguing possibility that the disease course notably in inflammatory pathology of the brain, may be different in the absence of the normally observed, concomitant upregulation of microglial PBR/TSPO. Immune-modulatory functions of the PBR/TSPO have previously been suspected based on pharmacological studies using PBR/TSPO binding compounds that appeared to abrogate inflammatory responses^21^,^22^,^23^.

Earlier work in bacteria^24^ suggests other functions than cholesterol transport for the PBR/TSPO, such as oxygen sensing and thus co-regulation of mitochondrial ATP production^25^, which may become relevant under a range of disease conditions. Possible functional links of the TSPO to the F_o_ sub-unit of the ATP synthase through the VDAC on the outer and ANT on the inner mitochondrial membrane have been described^25^,^26^.

Perceivably, PBR/TSPO-mediated changes in ATP production might explain indirect regulatory effects on the energy-dependent steroid biogenesis^1^,^3^,^10^, particularly under stress challenges. However, this alternative explanation of a functional association of the PBR/TSPO with steroid biogenesis necessitates re-examination of a substantial body of published research to separate direct from indirect or from non-selective, off-target effects. Many studies use compounds with purported selectivity for the PBR/TSPO for diagnostic or therapeutic purposes, such as those modulating complex behaviour in the apparent absence of binding to neural receptor systems^7^. Our study paradigmatically demonstrated for a long-established as well as a recently developed PBR/TSPO compound, that is, PK11195 and CLINDE/PBR111, respectively, that the *Guwiyang Wurra Tspo*^*−/−*^ strain is a powerful tool to discern selective from non-selective off-target binding and thus validate the purported drug actions in live animals under naturalistic conditions. We also anticipate innovative lipid membrane studies into the biophysical mechanisms by which ligands for the PBR/TSPO may either modify or introduce additional, potentially non-receptor, actions in the presence or absence of the protein^1^,^27^,^28^.

Lastly, the apparent irrelevance of the PBR/TSPO for the normal activation of microglia after neuronal injury indicates that neuro-glial interactions and inflammatory tissue responses are separate processes, though *in vivo* neuroimaging studies on both may regularly show high levels of PBR/TSPO expression^13^,^14^. The current concepts of ‘neuroinflammation’ do not adequately distinguish these and, therefore, too, merit revisiting.

The extensively characterized *Guwiyang Wurra Tspo*^*−/−*^ strain provides the broader research community with a means of dissecting the network of pathways through which the PBR/TSPO exerts its regulatory influence at the intersection of cell metabolism, innate immune response and endocrine regulation of behaviour in health and disease.

## Methods

### Generation of *Tspo* ^*−/−*^ mice

*Tspo* knockout mice were generated using a targeting construct that contained *lox*P sites flanking exons 2 and 3, and a neomycin cassette inserted between exons 3 and 4. The targeting construct was electroporated into C57BL/6 Bruce4 embryonic stem (ES) cells and cells correctly targeted by homologous recombination were injected into albino C57BL/6 blastocysts. Male chimeras were mated to female albino C57BL/6 mice and the resulting offspring with a black coat were screened for the presence of a targeted *Tspo* allele.

Mice positive for the presence of the targeted allele were crossed with C57BL/6 Cre deleter mice to remove the neomycin cassette and exons 2 and 3 to create heterozygous global *Tspo* deficient mice. To remove the Cre transgene, animals were bred to wild-type C57BL/6 mice.

To produce animals for experiments, heterozygous mice were crossed to generate wild-type littermate controls. All animal procedures were approved by the University of Sydney Animal Ethics Committee and the ANSTO Animal Care and Ethics Committee. The *Tspo*^*−/−*^ mouse strain has been given the additional designation *Guwiyang Wurra* (‘fire mouse’ in the local Dharawal language). Future naming will thus be C57BL/6-*Tspo*^*tm1GuMu(GuwiyangWurra)*^.

Mice were genotyped by Southern blot analysis using genomic DNA isolated from tail biopsies. For routine genotyping, genomic DNA was isolated from tail biopsies, ear biopsies or stool samples and amplified by PCR to generate a 489-bp product for the wild-type allele and a 246-bp product for the knockout allele. See Supplementary Table 4 for primer sequences. The PCR consisted of an initial incubation at 95 °C for 2 min, followed by 4 cycles at 95 °C for 30 s, 68 °C for 30 s and 72 °C for 2 min, then 4 cycles at 95 °C for 30 s, 65 °C for 30 s and 72 °C for 2 min, then 30 cycles at 95 °C for 30 s, 62 °C for 30 s and 72 °C for 2 min, and a final step at 72 °C for 5 min.

### Immunoblotting

Lysates (20 μg protein) were mixed with 2 × Laemmli sample buffer (Bio-Rad, Hercules, CA, USA), heated to 70 °C for 10 min and resolved on a 4–20% Mini-PROTEAN TGX gel (Bio-Rad). MagicMark XP (Life Technologies, Carlsbad, CA, USA) was used as the molecular weight marker. Proteins were transferred to a nitrocellulose membrane using the Trans-Blot Turbo transfer system (Bio-Rad). The membrane was incubated with anti-TSPO antibody (#109497, Abcam, Cambridge, UK) diluted 1:10,000 or anti-GAPDH (#37168, Abcam) diluted 1:1,000 and peroxidase-conjugated anti-rabbit antibody (#A0545, Sigma-Aldrich, St Louis, MO, USA) diluted 1:10,000. Pierce ECL Western Blotting Substrate (Thermo Scientific, Rockford, IL, USA) was used and the membrane visualized using an ImageQuant LAS 4000 (GE Healthcare, Little Chalfont, Buckinghamshire, UK).

### RT-PCR and quantitative real-time PCR

Tissue samples were homogenised in TRIzol (Life Technologies) and total RNA isolated using the PureLink RNA mini kit (Life Technologies) with on-column DNase treatment following the manufacturer’s instructions. Purified RNA (1 μg) was reverse-transcribed to cDNA using the SuperScript III First-Strand Synthesis kit (Life Technologies). For RT-PCR, cDNA from the kidneys of *Tspo*^*+/+*^ and *Tspo*^*−/−*^ mice was amplified using primers (Supplementary Table 4) located in exons 1 and 4. The PCR consisted of an initial incubation at 95 °C for 2 min, followed by 34 cycles at 95 °C for 30 s, 55 °C for 30 s and 72 °C for 1 min, and a final step at 72 °C for 5 min. The PCR products were confirmed by sequencing. For quantitative real-time PCR, cDNA was added to 2.5 μl of SsoFast EvaGreen Supermix (Bio-Rad) and 0.5 μM of each primer (Supplementary Table 4) in a final reaction volume of 5 μl. Reactions were performed on a CFX 384 Real-Time PCR detection System (Bio-Rad) by cycling at 98 °C for 30 s, followed by 45 cycles at 98 °C for 5 s and 63 °C (61 °C for the *Tspo2* assay) for 10 s. A melt curve analysis was performed to confirm the specificity of each reaction. Each sample was run in duplicate. The *Tspo2* primers were designed using Primer-BLAST and the specificity confirmed by DNA sequencing.

### Protoporphyrin IX

Blood from euthanized mice was placed into heparin tubes on ice, plasma removed, cells washed twice in ice-cold phosphate-buffered saline (PBS) and then resuspended in PBS to the original volume. Samples were evenly split (300 μl each), one receiving 1 mM 5-ALA and the other sterile H_2_O, and incubated in a shaking incubator at 37 °C for 4 h. Ethyl acetate/acetic acid (4:1) was added to each sample, then mixed and centrifuged before transferring the supernatant (1 ml) to a new tube with 1 ml of 1.5 M HCl. An aliquot of the lower HCl phase, after mixing and centrifuging the sample, was measured in a spectrofluorimeter (excitation wavelength 407 nm, slit width 10 nm, emission spectrum 450–800 in 1 nm increments). Glass tubes and containers were used throughout and samples were protected from light.

### Facial nerve axotomy

Facial nerve axotomy^29^,^30^ was performed on *Tspo*^*+/+*^ and *Tspo*^*−/−*^ mice (*n*>4 per genotype) anaesthetized with 5% (v/v) isoflurane. A small incision (0.5–0.7 cm) in the skin 0.5 cm dorsal and lateral to the ear was made and the facial nerve transected under an operating microscope. The incision was closed with Vetbond Tissue Adhesive (3M, St Paul, MN, USA). The success of the operation was confirmed by the loss of ipsilateral whisker movement. Mice were monitored carefully and euthanized with CO_2_ 3 days after the operation. Brains were dissected, placed in OCT, transferred to liquid nitrogen and stored at −80 °C until cryo-sectioning.

### Stereotactic implantation of GL261 mouse glioblastoma cells

The *Tspo*^+/+^ (*n*=6) and *Tspo*^*−/−*^ mice (*n*=4) were operated under isofluorane anaesthesia and aseptic conditions using a Kopf stereotactic apparatus and precision dental drill (Kopf Instruments, Tujunga, CA, USA). A small burr hole was made 3 mm anterior of the bregma and 2 mm lateral over the right frontal lobe (3 mm ventral). GL261 glioblastoma cells (NCI, Bethesda, MD, USA) at 3 × 10^3^ per μl in a volume of 10 μl were slowly injected over 2 min using a 10-μl Hamilton syringe connected to a pump by means of a 32-gauge needle. The animals were closely monitored daily for good recovery, including body weight and absence of neurological signs. Three and four weeks after implantation of the GL261 cells, the animals were imaged by means of microPET/CT and brains were dissected for histological studies and autoradiography.

### PET and CT imaging

Mice, anaesthetized (5% (v/v) with isoflurane and maintained at 1–2%, were scanned using a small-animal Inveon PET/CT scanner (Siemens, Knoxville, TN, USA) according to the methods described previously^31^,^32^. Body temperature was maintained with a feedback regulated heating pad and respiration monitored (BioVet; m2m Imaging Corp, Cleveland, OH, USA). Scans started with the tail vein injection of [^18^F]PBR111 (8–18 MBq per 100 μl, 0.2 nM). After 40 min of imaging, PBR111 (1 mg per kg in 2% acetic acid–saline) was injected to determine non-specific accumulation in organs and imaged for 10 min. Then, mice underwent a 10-min CT scan for anatomical information. All PET data were corrected, normalized and reconstructed with an OSEM3D–MAP algorithm^31^ to produce PET volumes of activity concentration (kBq per ml).

### Autoradiography

Tissue sections from *Tspo*^*+/+*^, *Tspo*^+/−^ and *Tspo*^*−/−*^ mice were incubated at room temperature for 20 min in 170 mM Tris-HCl pH 7.4 containing 1 nM [^3^H]PK11195 (specific activity 84 Ci per mmol; Perkin Elmer, Waltham, MA, USA), washed twice for 5 min in 170 mM Tris-HCl, rinsed with 3 dips in ice-cold MilliQ H_2_O, and dried. Additional sections were incubated on ice for 1 h in 50 mM Tris-HCl pH 7.4 containing 3 nM [^125^I]CLINDE (specific activity 100 Ci per mmol; synthesized by ANSTO Life Sciences), washed twice for 2 min in ice-cold 50 mM Tris-HCl, for 1 min in ice-cold MilliQ H_2_O, and dried. Displacement binding was carried out in the presence of PK11195 (10 μM), CLINDE (10 μM) and PBR111 (10 μM). Single-emulsion films were exposed to sections and standards (^14^C or ^3^H) for 3 h ([^125^I]CLINDE) or 10 weeks ([^3^H]PK11195).

### Immunohisto- and cytochemistry

Immunohistochemistry was performed as previously described^13^,^33^,^34^. Tissue sections (used for autoradiography) were fixed with 3.7% formaldehyde in PBS for 5 min, then permeabilized with ice-cold acetone. Non-specific antibody binding was blocked with 10% horse serum and 2% bovine serum albumin (BSA) in PBS. Sections were incubated with TSPO monoclonal antibody (#109497, Abcam) at 4 °C overnight and secondary HRP-conjugated goat anti-rabbit antibody at RT for 1 h (#A0545, Sigma-Aldrich). The activity of HRP was detected with the peroxidase 3,3′-diaminobenzidine tetrahydrochloride liquid substrate system (Sigma-Aldrich). Sections were dehydrated with ethanol, incubated with xylene, and slides were mounted in DPX mounting media with a coverslip. Sections were visualized under an inverted Olympus BX51 microscope (Olympus, Tokyo, Japan), and captured with a Q-imaging camera and ImagePro 5.1 program.

A Zeiss LSM 710 confocal microscope with a 5 × EC Plan-Neofluar NA 0.16 objective was used for the fluorescence microscopy of the facial nucleus. Alexa Fluor 568 was excited with a 561-nm laser, using a 488/561/633 dichroic mirror, and emission captured from 565 to 640 nm.

For immunocytochemistry, peritoneal macrophages were isolated according to Zhang *et al*.^35^. After 2 days in complete cultural medium the purity of macrophages was >97% as confirmed with the macrophage-specific marker IB4 conjugated with FITC (Sigma-Aldrich). Cells on coverslips were fixed and incubated using the same method as for tissue sections, with the addition of a mouse anti-human/mouse mitochondrial electron transport chain complex IV antibody (#AB14705, Abcam), then incubated with the secondary antibodies Alexa Fluor (AF) 594-conjugated goat anti-rabbit antibody (Life Technologies) and AF488-conjugated goat anti-mouse antibody (Life Technologies). Cells on coverslips were mounted on microscope slides with ProLong Gold antifade reagent containing DAPI (Life Technologies) and viewed under a BX61WI Olympus microscope. Images were acquired with a digital camera (CoolSNAP, Photometrics, Tucson, AZ, USA) and the Image InVivo program (Photometrics). Deconvolution of images was performed with the AutoDeblur program (Photometrics) and further processed with ImageJ (NIH, Baltimore, MD, USA).

### Primary microglial cell culture

Microglia were cultured from 0–2-day-old *Tspo*^*+/+*^ and *Tspo*^*−/−*^ mice according to methods described previously^33^. Briefly, whole brains were dissected, cut into fine pieces and treated with 0.025% trypsin (Sigma-Aldrich). They were cultured in Dulbecco’s modified Eagle’s medium (DMEM/F12; Sigma-Aldrich) supplemented with 10% fetal bovine serum (FBS; Life Technologies), 1% penicillin-streptomycin-glutamine (Sigma-Aldrich) and 0.5 ng per ml GM-CSF (Abcam).

Microglia were purified by shaking at 350 revolutions per minute (r.p.m.) for 50 min at 37 °C, pelleted by centrifugation, resuspended in supplemented DMEM/F12 and placed (4 × 10^4^ cells per well) into a 96 well Seahorse XF cell culture plate (Seahorse Bioscience, North Billerica, MA, USA) pre-coated with FBS for 15 min at room temperature. After 15 min the wells were washed twice to remove unattached cells. The purity of microglial cultures was >98% as confirmed by staining with the microglial marker Alexa Fluor-conjugated isolectin GS-IB4 (Life Technologies). The 5 × 10^4^ microglia from wild-type and knockout mice were plated into each well of the XF96 plate and were grown for 2–3 days before evaluating mitochondrial functions with the Seahorse XF 96 Cell Analyzer (Seahorse Bioscience). To rule out systematic differences in cell density and viability between *Tspo*^*+/+*^ and *Tspo*^*−/−*^ microglia, cell density was monitored and assured throughout the experimental measurements, while viability, including apoptosis, was confirmed using caspase 3/7 (ref. 36).

### Measurements of mitochondrial respiration

The oxygen consumption rate (OCR) and extracellular acidification rate in primary microglial cultures were determined using the Seahorse XF 96 Cell Analyzer. OCR is an indicator of mitochondrial respiration^37^,^38^. The day before the experiment 200 μl of the Seahorse XF Calibrant (pH 7.4) was added to each well of the XF 96-well utility plates. The sensor cartridge was placed on top of the plate and hydrated for 16 h at 37 °C in a non-CO_2_ incubator. The protocol for basal measurements of the cells at 37 °C (start, calibrate probes, equilibrate, mix 2 min, 1 min delay, measure 3 min) was repeated twice. Mitochondrial stress compounds (oligomycin, rotenone combined with antimycin A) were then injected, followed by 2 min mixing, 1 min delay, measure 3 min (repeated 3 times).

On the day of the experiment, microglia (plating density 5 × 10^5^ per well, purity >98% and viability >92% for both wild-type and knockout microglia) were washed 3 times with un-buffered DMEM plus 20 mM glucose (no buffers) and incubated at 37 °C (no CO_2_) for 1 h. Oligomycin and rotenone combined with antimycin A were used to determine mitochondrial ATP production and basal mitochondrial OCR^37^,^38^. Oligomycin and the combination of rotenone and antimycin A were injected at concentrations of 0.3, 1 and 3 μM (optimal for oligomycin) and 10 μM (optimal for rotenone and antimycin A).

The data for each reported parameter reflect 11–14 independent Seahorse XF measurement series from a total of seven independently repeated microglial cell culture preparations.

### Radioligand binding

Organs were homogenized using a T25 digital Ultra-Turrax homogenizer (Ika, Wilmington, NC, USA) at speed setting 5 and 20,000 r.p.m. in ~45 ml of ice-cold TRIS buffer (pH 7.4) and washed twice in ice-cold 50 mM Tris-HCl (pH 7.4) by centrifugation at 48,000 *g*, resuspended in 50 volumes of ice-cold 50 mM Tris-HCl, and protein concentration was determined using a BCA protein assay kit (Thermo Scientific). Bmax and Kd were determined with saturation binding by incubating membrane protein (60 μl) with [^3^H]PK11195 (0.56–20 nM) on ice for 90 min before harvesting by rapid filtration through Whatman GF/C filters (GE Healthcare) presoaked in a 0.5% polyethylenimine solution. Non-specific binding was determined with 5 μM PK11195. Filters were placed in scintillation cocktail (Perkin Elmer) for 12 h and radioactivity was determined using a Tri-Carb 2100 TR Liquid Scintillation Counter (Perkin Elmer). Bmax and Kd values were obtained by non-linear regression using GraphPad Prism 5.04 (GraphPad Software, La Jolla, CA, USA) by fitting total and non-specific binding.

### Cholesterol transport and biosynthesis

P450scc metabolism was measured in mouse testis according to Tuckey *et al*.^39^ and Slominski *et al*.^40^. Briefly, an enriched mitochondrial fraction was prepared by centrifugation in 250 mM sucrose, 50 mM Tris (pH 7.4), and the enzymatic conversion of cholesterol to pregnenolone was conducted under the following conditions out to a maximum 2-h time-point: 50 mM HEPES pH 7.4, 250 mM sucrose, 20 mM KCl, 5 mM MgSO_4_, 0.2 mM EDTA, 1 mg per ml BSA, 8 μM Trilostane, 0.05 μCi ^3^H-cholesterol, 500 mM isocitrate and 5 mM NaNADP. Reactions were terminated with the addition of 4 °C dichloromethane. The organic phase was retained and the aqueous phase was re-extracted twice more into dichloromethane. Extracts were combined, dried under nitrogen and dissolved in 100 μl of ethyl acetate. Pregnenolone and cholesterol were separated by thin-layer chromatography, with hexane:diethylether:acetic acid (15:15:1) mobile phase, and the amount of radioactivity in each spot quantified by liquid scintillation counting.

### Detection of serum pregnenolone

Serum pregnenolone of male and female *Tspo*^*+/+*^ and *Tspo*^*+/+*^mice was determined by ELISA performed according to the manufacturer’s instructions (Abnova Cooperation, Taiwan). Briefly, blood was collected, allowed to clot, and the serum collected. Standards, controls and samples were incubated in duplicate in a sealed plate with or without conjugate and antibody for 1 h at room temperature on a plate shaker (KS4000ic, IKA, Selangor, Malaysia) at 200 r.p.m. After the incubation, wells were emptied, washed five times, and the plate tapped on lint free paper towel to remove any remaining buffer. Wells were incubated with HRP conjugate on the plate shaker at 200 r.p.m. for 30 min at room temperature, washed five times, and incubated with TMB substrate for 10 min at room temperature. Stop solution was added to each well and after 20 min the optical density at 450 nm was measured. Data were normalized against blood volume and analysed using a four-parameter logistic curve fitting program MasterPlex ReaderFit: Curve-Fitting Software for ELISA Analysis (Hitachi Solution America, San Francisco, CA, USA).

### Blood phenotyping

Thirty mice (10 *Tspo*^*+/+*^, 10 *Tspo*^+/−^, 10 *Tspo*^*−/−*^, 5 months old, males/females evenly distributed) used in behavioural experiments were euthanized with CO_2_. Blood was collected from the pulmonary cavity by cutting the inferior vena cava and placed in microtubes with heparin (haematology and lymphocyte analysis) or gel clot activator (biochemical analysis). Spleens were collected in 10 ml of ice-cold sterile PBS. Blood (room temperature) and spleens (on ice) were delivered (within 3–7 h of collection) to the Australian Phenomics Facility for flow cytometric and biochemical analysis.

For general haematology, blood was diluted 1:2 and analysed with fluorescence-activated cell sorting using the Advia 2120 Haematology System (Siemens, Munich, Germany). Blood cell subsets, including white blood cells and platelets, were assessed. For lymphocyte analysis, blood was analysed for the presence or absence of abnormalities in T cells, B cells, NK cells and monocytes. The T-cell subsets included CD4+ and CD8+ subsets, while the B-cell subsets included immature and mature B cells and IgE+ monocytes. Spleens were analysed by flow cytometry and stained for NK cells, for B cells, including mature and immature subsets, and for T cells, including CD4+ and CD8+ cells and their activated subsets. See Supplementary Table 3 for a list of cell types examined.

For biochemistry, blood was centrifuged, the serum collected and run through the Olympus AU400 Chemistry Analyzer (Olympus), which detects levels of cholesterol, triglycerides, glucose, high-density lipoprotein (HDL), albumin, creatine kinase activity and alanine aminotransferase activity. See Supplementary Table 3 for a list of biochemical parameters examined.

### Mouse sperm analysis

Sperm were released from the cauda epididymides of *Tspo*^*+/+*^ and *Tspo*^*−/−*^ mice by cutting each cauda with a scalpel and incubating in 150 μl of pre-warmed Biggers–Whitten–Whittingham (BWW) medium for 5 min at 37 °C. After the incubation, an additional 250 μl of BWW medium was added, the tissue removed and the sperm assessed using a computer-assisted sperm analysis system (Hamilton Thorne, Beverly, MA, USA). Parameters assessed were total motile, progressive motile, VAP, VSL and VCL.

### Assessment of anxiety-related behaviours

Behavioural tests were performed on mice aged between 3–4 months, handled regularly to reduce the impact of handling stress during testing. Anxiety-related behaviours were examined with the following: open-field test, emergence test, light/dark preference test and elevated plus maze. In each test the mice were allowed free exploration in the apparatus for 15 min. The duration and frequency of entering each compartment, total distance travelled, time spent active and the number of stool droppings were assessed. In the elevated plus maze risk assessment was defined as when an animal’s head was facing towards an open arm with more than 15% of its body in an open arm while maintaining its centre of mass within the closed arm or central platform. The genotype of the animals was blind to the experimenter during testing. Tests were carried out at least 7 days apart to reduce the effects of training history^41^. All experiments were recorded with an overhead camera on to DVD and later analysed using Motman Tracker 4.5 software (Motion Mensura, Cooks Hill, NSW, Australia).

### Data analysis

Statistical significance was calculated using an unpaired two-tailed Student’s *t*-test for two-group comparisons. For comparisons of more than two groups univariate analysis of variance (ANOVA) with a Bonferroni’s *post-hoc* test was performed and considered significant at *P*≪0.05. Data are presented as mean±standard deviation, except where indicated otherwise.

## Author contributions

R.B.B., G.-J.L. and R.J.M. designed the knockout. G.-J.L. and R.J.M. performed most of the experiments. R.C. contributed animal behaviour, autoradiography, radioligand membrane receptor binding studies and analysis of blood phenotyping. C.R.H. contributed autoradiography and immunohistochemistry. W.W.-Y.K. contributed autoradiography and RT-qPCR. C.Q. contributed cell culture and measurement of mitochondrial respiration. R.B.B. and M.B.G. performed facial nerve axotomy, histological, immunofluorescence staining, as well as the brain tumour implant experiments. S.F. undertook the confocal microscopy of the facial nucleus. A.P., D.Z., P.C., M.G. and A.S. contributed PET imaging. T.P. provided [^18^F]PBR111 and [^125^I]CLINDE. N.R.H. performed the cholesterol transport and biosynthesis experiments. R.B.B. and G.-J.L. devised and supervised the project. R.B.B. wrote the manuscript with assistance by G.-J.L., R.J.M. and C.R.H.

## Additional information

**How to cite this article:** Banati, R. B. *et al*. Positron emission tomography and functional characterization of a complete PBR/TSPO knockout. *Nat. Commun.* 5:5452 doi: 10.1038/ncomms6452 (2014).

## Supplementary Material

Supplementary Information Supplementary Figures 1-3, Supplementary Tables 1-4 and Supplementary References
