# Supplementary material for: Positron emission tomography and functional characterization of a complete PBR/TSPO knockout
Source: Nat Commun. 2014 Nov 19;5:5452. doi: 10.1038/ncomms6452 (PMC4263137; doi:10.1038/ncomms6452)
Supplement: Supplementary Information — Supplementary Figures 1-3, Supplementary Tables 1-4 and Supplementary References [file ncomms6452-s1.pdf]

Supplementary Information:

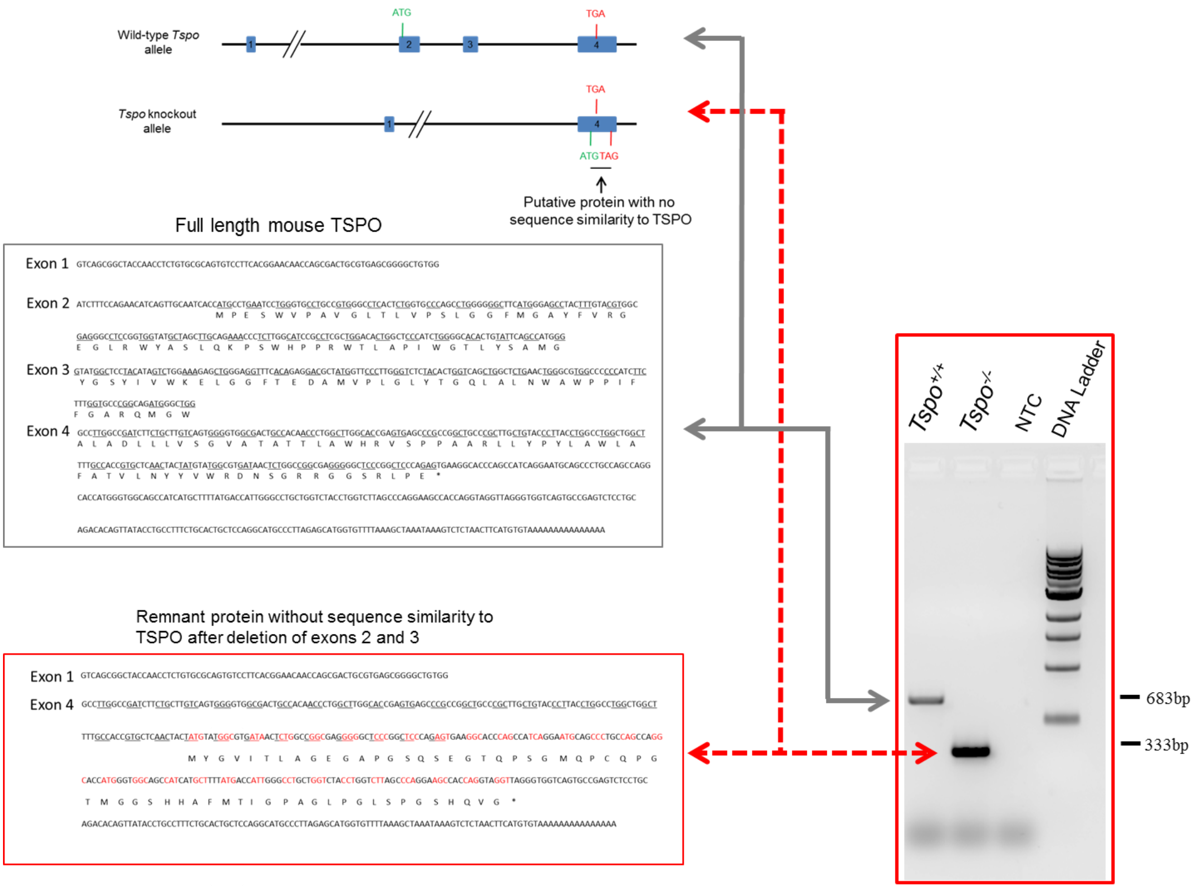

Supplementary Figure 1. Confirmation of the deletion of exons 2 and 3.

By deleting exons 2 and 3, only exons 1 and 4 remain. As can be observed in the above sequence of the full length *Tspo* gene, there are no start codons within the *Tspo* reading frame present in either exon 1 or exon 4. Therefore, following the deletion of exons 2 and 3, only a short protein that shares no sequence similarity to the TSPO, as shown above, could in theory be produced. The presence of a mRNA sequence corresponding to exons 1 and 4 in *Tspo* knockout mice was confirmed by RT-PCR. The cDNA from a *Tspo* knockout animal produced a product of the expected size (333 bp; red dotted line), whilst the cDNA from a wildtype animal produced a product of 683 bp which corresponds to exons 1, 2, 3 and 4. The products from both wildtype and *Tspo* knockout animals were confirmed by DNA sequencing.

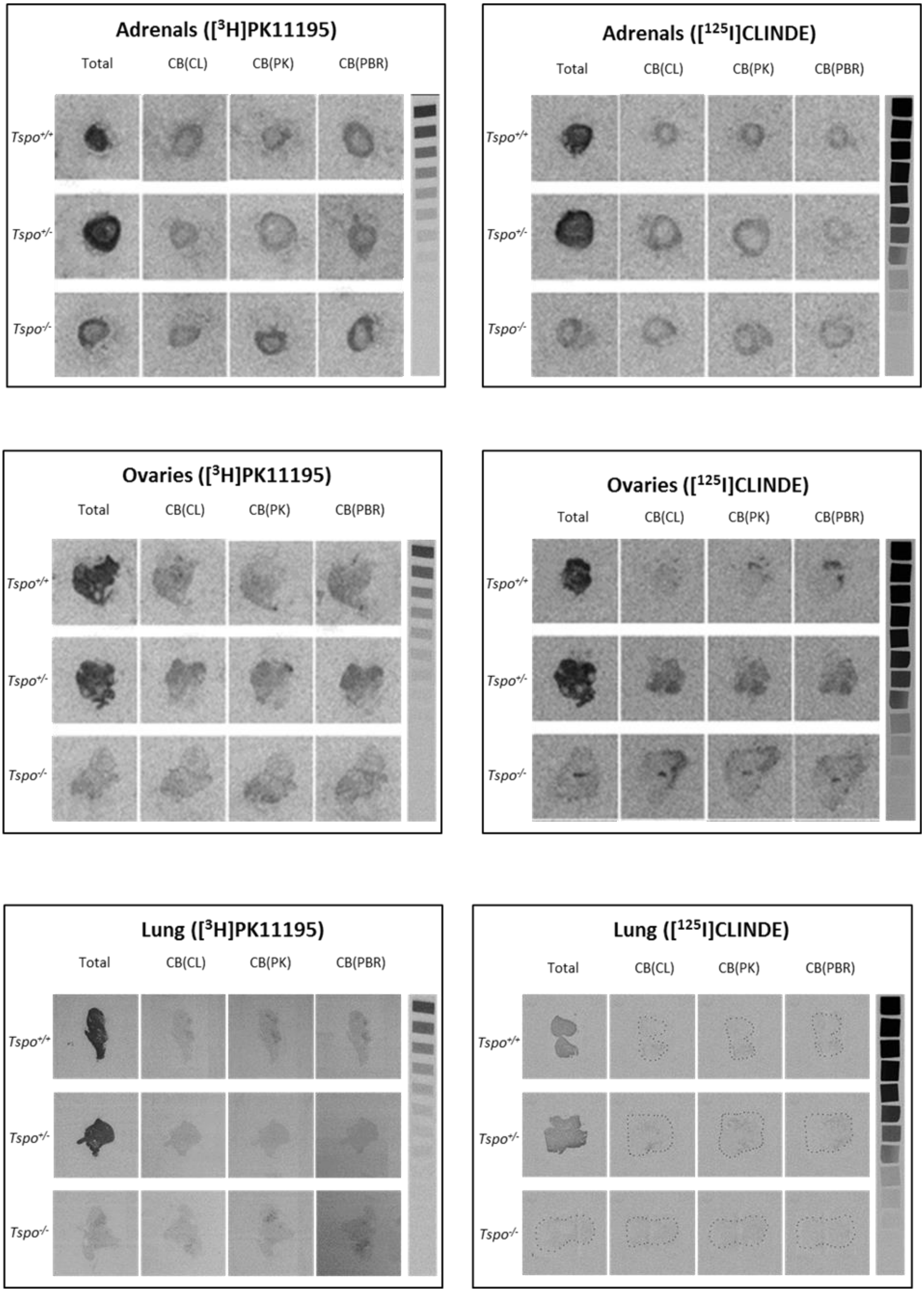
Adrenals ([3H]PK11195)
Total
CB(CL)
CB(PK)
CB(PBR)
Tspo+/+
Tspo+/-
Tspo-/-
Adrenals ([125I]CLINDE)
Total
CB(CL)
CB(PK)
CB(PBR)
Tspo+/+
Tspo+/-
Tspo-/-
Ovaries ([3H]PK11195)
Total
CB(CL)
CB(PK)
CB(PBR)
Tspo+/+
Tspo+/-
Tspo-/-
Ovaries ([125I]CLINDE)
Total
CB(CL)
CB(PK)
CB(PBR)
Tspo+/+
Tspo+/-
Tspo-/-
Lung ([3H]PK11195)
Total
CB(CL)
CB(PK)
CB(PBR)
Tspo+/+
Tspo+/-
Tspo-/-
Lung ([125I]CLINDE)
Total
CB(CL)
CB(PK)
CB(PBR)
Tspo+/+
Tspo+/-
Tspo-/-

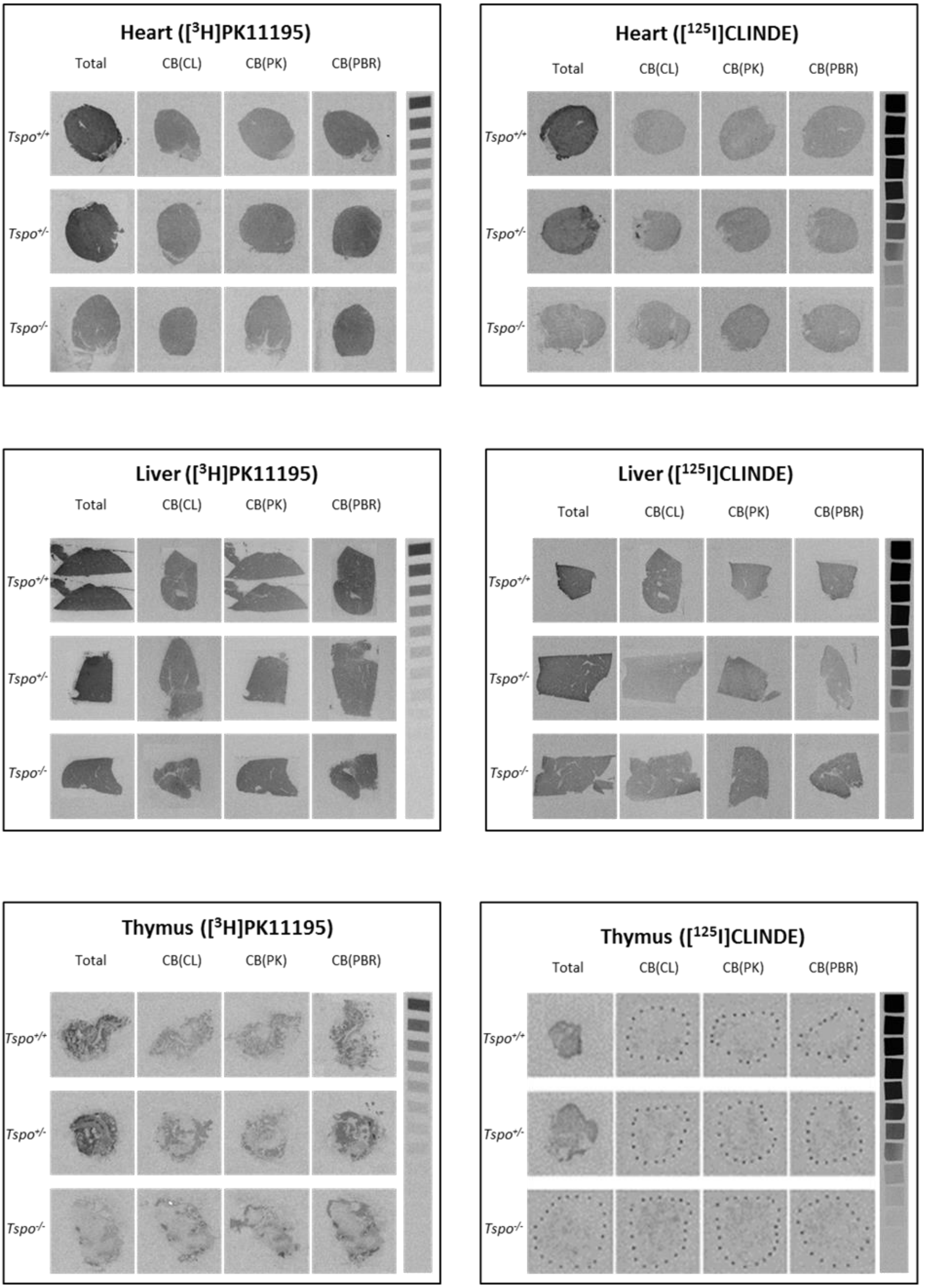
Heart ([3H]PK11195)
Total
CB(CL)
CB(PK)
CB(PBR)
Tspo+/+
Tspo+/-
Tspo-/-
Heart ([125I]CLINDE)
Total
CB(CL)
CB(PK)
CB(PBR)
Tspo+/+
Tspo+/-
Tspo-/-
Liver ([3H]PK11195)
Total
CB(CL)
CB(PK)
CB(PBR)
Tspo+/+
Tspo+/-
Tspo-/-
Liver ([125I]CLINDE)
Total
CB(CL)
CB(PK)
CB(PBR)
Tspo+/+
Tspo+/-
Tspo-/-
Thymus ([3H]PK11195)
Total
CB(CL)
CB(PK)
CB(PBR)
Tspo+/+
Tspo+/-
Tspo-/-
Thymus ([125I]CLINDE)
Total
CB(CL)
CB(PK)
CB(PBR)
Tspo+/+
Tspo+/-
Tspo-/-

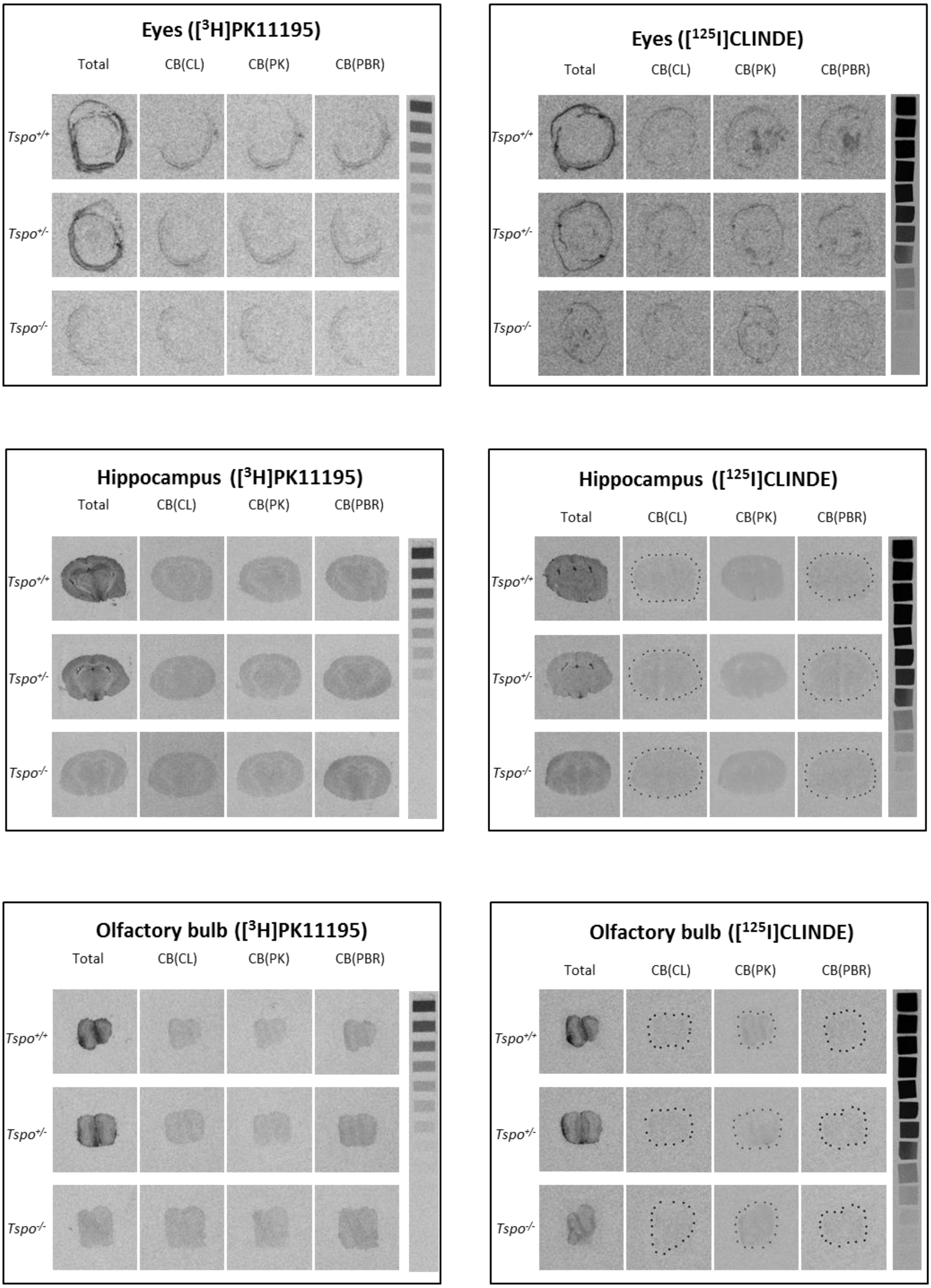
Eyes ([3H]PK11195)
Eyes ([125I]CLINDE)
Hippocampus ([3H]PK11195)
Hippocampus ([125I]CLINDE)
Olfactory bulb ([3H]PK11195)
Olfactory bulb ([125I]CLINDE)
Total
CB(CL)
CB(PK)
CB(PBR)
Tspo+/+
Tspo+/-
Tspo-/-

Supplementary Figure 2. Comparative [^{3}H]PK11195 and [^{125}I]CLINDE autoradiography of 9 organs in adult mice.

The receptor autoradiographs (16 µm sections) show the total binding of [^{3}H]PK11195 (1 nM) and [^{125}I]CLINDE (3 nM) as well as competitive displacement with 10 µM unlabelled CLINDE (CB(CL)), PK11195 (CB(PK)) and PBR111 (CB(PBR)) ofadrenal glands, ovaries, lungs, heart, liver, thymus, eyes, hippocampus and olfactory bulb of *Tspo*$^{+/+}$, *Tspo*$^{+/-}$ and *Tspo*$^{-/-}$ mice .

Specific binding of 3 nM [^{125}I]CLINDE and 1 nM [^{3}H]PK11195 is clearly visible in tissue sections from *Tspo*$^{+/+}$ and *Tspo*$^{+/-}$ mice and is displaceable by all 3 unlabelled ligands, whereas there is no specific binding in *Tspo*$^{-/-}$ tissue.

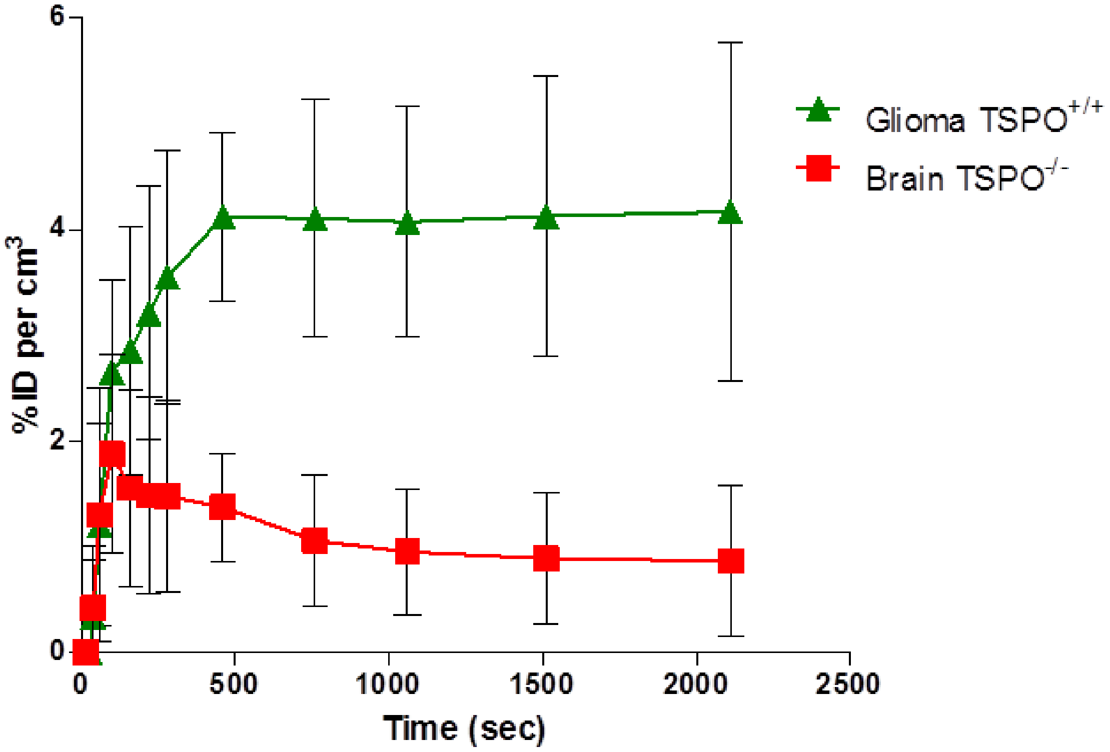

Supplementary Figure 3. Quantification of [^{18}F]PBR111 positron emission tomography of PBR/TSPO-expressing glioma in global TSPO knockout mouse brain.

Time-activity curves of [^{18}F]PBR111 25 days after GL261 glioblastoma cell implantation. (green = *Tspo*$^{+/+}$ glioma*; red* = *Tspo*$^{-/-}$ brain). Error bars denote standard deviation (n=4 for each group).

Supplementary Table 1. Diet Composition

Calculated Nutritional Parameters	Premium bredder diet - Gordon's Specialty Stockfeeds	Calculated Total Vitamins	Premium bredder diet - Gordon's Specialty Stockfeeds
Protein	23%	Vitamin A (Retinol)	5666 IU/kg
Total Fat	6%	Vitamin D (Cholecalciferol)	200 IU/kg
Crude Fibre	5%	Vitamin E (a Tocopherol acetate)	50 mg/kg
Metabolizable energy	13 MJ/ kg	Vitamin K (Menadione)	5 mg/kg
**Calculated Amino Acids**		Vitamin C (Ascorbic acid)	150 mg/kg
Arginine	1.21%	Vitamin B1 (Thiamine)	4 mg/kg
Valine	1.02%	Vitamin B2 (Riboflavin)	5 mg/kg
Leucine	1.52%	Niacin (Nicotinic acid)	10 mg/kg
Isoleucine	0.80%	Vitamin B6 (Pryridoxine)	6 mg/kg
Threonine	0.84%	Pantothenic Acid	12 mg/kg
Methionine	0.59%	Biotin	0.06 mg/kg
Cystine		Folic Acid	10 mg/kg
Lysine	0.98%	Vitamin B12 (Cyancobalamin)	5 µg/kg
Phenylalanine	1.64%	**Calculated Fatty Acid Composition**	
Tyrosine		Total Mono Unsaturated Fats	42.90%
Tryptophan	0.37%	Total Polyunsaturated Fats	30.70%
Histidine	0.50%	Total Saturated Fats	21.30%
**Calculated Total Minerals**			
Calcium	1.01%		
Phosphorous	0.77%		
Magnesium	0.18%		
Sodium	0.30%		
Potassium	0.54%		
Iron	97.0 mg/kg		
Copper	10.6 mg/kg		
Iodine	1.15 mg/kg		
Manganese	87.4 mg/kg		
Zinc	48.1 mg/kg		
Selenium	0.1 mg/kg		

Supplementary Table 2. Behaviour phenotyping

Test	index	gender	TSPO$^{+/+}$	TSPO$^{+/-}$	TSPO$^{-/-}$
Open field	Time spent in center (sec)	Male<br>Female	160.1±101.1<br>94.33±24.28	87.70±36.35<br>124.0±45.96	112.3±43.01<br>99.94±33.94
	Frequency of entering center	Male<br>Female	60.57±23.78<br>48.83±10.89	49.75±16.61<br>53.75±14.83	53.42±10.40<br>58.64±13.92
	Time spent active (sec)	Male<br>Female	711.0±50.49<br>714.3±31.12	704.0±53.59<br>676.4±42.13	738.0±26.08<br>725.7±38.34
	Distance travelled (mm)	Male<br>Female	43180±11069<br>44266±7782	44381±10006<br>39516±10048	42084±7590<br>50333±12334
	Distanced moved without travelling (mm)	Male<br>Female	4308±758.1<br>4092±584.8	4185±739.5<br>3729±476	4693±556.8<br>4444±603.8
Emergence test	Time spent in hidebox (sec)	Male<br>Female	155.4±42.56<br>121.8±41.59	129.1±35.64<br>113.1±44.85	159.8±56.15<br>121.1±43.18
	Frequency of entering hidebox	Male<br>Female	30.28±15.22<br>25.17±9.411	25.55±6.629<br>23.00±10.50	26.42±9.020<br>28.55±9.903
	Time spent active (sec)	Male<br>Female	751.9±25.91<br>705.7±38.97	746.2±31.06<br>711.9±35.08	752.1±32.88<br>748.3±32.77
	Distance travelled (mm)	Male<br>Female	44545±4826<br>41125±7451	45864±6128<br>42068±8191	41474±5881<br>49188±8525
	Distance moved without travelling (mm)	Male<br>Female	5765±528.4<br>4781±730.2	5675±673.2<br>4753±641.5	5631±784.8<br>5335±676.8
Light/dark preference test	Time spent in light compartment (sec)	Male<br>Female	382.1±115.7<br>418.2±84.50	422.8±112.5<br>372.0±85.95	374.9±76.04<br>412.9±113.0
	Transition between compartments	Male<br>Female	32.00±6.403<br>41.33±10.29	36.67±11.66<br>46.62±19.64	33.08±8.229<br>46.09±9.148
	Time spent active (sec)	Male<br>Female	819.4±25.79<br>803.8±20.20	821.7±15.86<br>797.0±19.08	827.5±12.60<br>810.5±17.62
	Distance travelled (mm)	Male<br>Female	50219±5948<br>53074±9692	52916±5955<br>48749±6220	50071±6780<br>58633±9674
	Distance moved without travelling (mm)	Male<br>Female	8363±943.2<br>7682±443.0	8367±790.1<br>7219±606.8	8359±532.8<br>7712±735.3
Elevated plus maze	Time spent in open arms (sec)	Male<br>Female	134.6±59.75<br>103.8±55.92	122.9±75.71<br>139.2±78.00	145.1±59.14<br>152.46±87.09
	Frequency of entering open arms	Male<br>Female	33.14±8.783<br>25.50±11.15	28.29±13.83<br>28.62±11.35	29.50±7.205<br>31.55±17.40
	Time spent active (sec)	Male<br>Female	646.9±42.39<br>607.8±74.20	619.0±63.45<br>595.6±64.95	623.3±36.55<br>624.5±62.23
	Distance travelled (mm)	Male<br>Female	21188±3554<br>17096±4673	18871±5954<br>17996±4994	19140±3803<br>20150±6317
	Distanced moved without travelling (mm)	Male<br>Female	5020±624.9<br>4737±726.9	4861±632.6<br>4679±607.2	4824±298.2<br>4974±550.3

No significant behavioural differences were found between genotypes tested with two-way ANOVA. Values represent mean ± standard deviation (n=5-10 per group).

Supplementary Table 3. Blood phenotyping

analysis	phenotype	gender	$TSPO^{+/+}$	$TSPO^{+/-}$	$TSPO^{-/-}$
Haematology	Red blood cells (RBC) ($x10^6$ cells/μL)	Male<br>Female	9.220±1.067<br>9.948±0.591	9.612±1.117<br>10.36±0.654	9.560±0.473<br>9.795±0.756
	White blood cell (WBC) ($x10^3$ cells/μL)	Male<br>Female	7.428±2.195<br>10.21±3.572	8.024±3.957<br>8.992±3.205	8.440±1.612<br>8.635±2.317
	Platelets ($x10^3$ cells/μL)	Male<br>Female	828.4±479.6<br>970.0±184.7	1032±302.8<br>846.0±423.3	1104±143.4<br>950.0±94.84
	Haematocrit (L/L)	Male<br>Female	0.454±0.061<br>0.486±0.038	0.460±0.052<br>0.480±0.027	0.464±0.021<br>0.472±0.039
	Reticulocytes ($x10^3$ cells/μL)	Male<br>Female	332.6±21.52<br>377.8±66.10	350.4±26.28<br>415.6±83.99	341.6±33.55<br>380.8±95.63
	Haemoglobin (g/L)	Male<br>Female	102.0±13.49<br>109.2±9.230	102.4±9.63<br>109.6±6.986	102.4±6.066<br>105.5±8.062
	Mean corpuscular volume (fL)	Male<br>Female	98.20±3.421<br>97.60±3.647	96.00±2.121<br>93.20±1.095	97.20±3.421<br>96.00±2.944
Biochemistry	Na^+ (mmol/L)	Male<br>Female	156.3±2.645<br>155.3±2.490	157.3±3.854<br>153.9±3.488	157.6±1.989<br>156.1±1.792
	K^+ (mmol/L)	Male<br>Female	8.040±0.744<br>7.060±0.770	7.675±0.704<br>6.850±0.645	7.620±0.349<br>7.160±0.550
	Cl^- (mmol/L)	Male<br>Female	112.6±0.894<br>111.2±0.837	112.0±0.816<br>112.0±2.160	111.0±1.871<br>113.4±2.881
	Glucose (mmol/L)	Male<br>Female	9.550±1.759<br>10.06±1.431	10.11±1.417<br>11.01±2.393	10.13±1.534<br>11.55±1.611
	Cholesterol (mmol/L)	Male<br>Female	2.404±0.706<br>2.512±0.622	2.707±0.701<br>2.967±0.085	2.894±0.458<br>2.324±0.671
	Triglyceride (mmol/L)	Male<br>Female	1.000±0.000<br>1.200±0.447	1.500±0.577<br>1.250±0.500	1.600±0.548<br>1.138±0.500
	High density lipoprotein (mmol/L)	Male<br>Female	1.800±0.837<br>1.800±0.447	1.750±0.500<br>2.000±0.000	2.000±0.000<br>1.632±0.507
	Creatine kinase (U/L)	Male<br>Female	1507±1254<br>1452±1047	2031±1339<br>3576±156.3	1067±766.8<br>6417±10647
	Alanine aminotransferase (U/L)	Male<br>Female	29.14±9.492<br>36.22±12.78	52.98±38.63<br>56.58±13.78	43.62±6.454<br>81.98±81.76
	Bilirubin (mmol/μL)	Male<br>Female	4.100±1.173<br>3.180±0.630	3.050±0.819<br>3.875±0.640	4.200±2.893<br>4.100±1.183
Lymphocyte FACs	Lymphocytes (%)	Male<br>Female	52.86±4.794<br>54.86±6.138	51.80±4.770<br>49.38±5.053	48.66±6.494<br>49.96±8.400
	T-cells (%)	Male<br>Female	22.56±5.854<br>21.12±4.395	19.90±2.554<br>18.66±4.873	18.94±2.214<br>20.46±5.793
	$CD4^+$ T-cells (%)	Male<br>Female	41.32±5.295<br>38.34±4.702	40.56±3.930<br>36.40±3.449	40.00±3.845<br>39.14±3.745
	Activated $CD4^+$ T-cells (%)	Male<br>Female	49.02±9.648<br>48.30±6.120	47.14±3.702<br>49.82±6.994	51.16±3.233<br>48.90±15.04
	$CD8^+$ T-cells (%)	Male<br>Female	33.68±3.328<br>34.10±1.612	33.74±3.702<br>32.54±5.192	34.46±2.370<br>32.04±4.922
	Activated $CD8^+$ T-cells (%)	Male<br>Female	36.74±8.442<br>29.78±5.325	36.20±7.563<br>30.68±9.696	36.16±6.690<br>33.92±6.479
	B-cells (%)	Male<br>Female	56.52±4.177<br>60.40±5.561	57.38±3.433<br>53.88±6.548	60.56±9.176<br>56.28±5.356
	Mature B-cells (%)	Male<br>Female	5.980±0.916<br>6.548±2.391	6.950±1.022<br>9.210±2.962	6.580±1.833<br>7.686±2.276
	Immature B-cells (%)	Male<br>Female	88.58±0.963<br>86.58±3.910	87.620±1.190<br>81.600±6.005	87.00±3.811<br>83.88±3.454
	Double negative B-cells (%)	Male<br>Female	4.610±0.313<br>6.116±1.396	4.692±0.504<br>8.316±2.958	5.668±1.996<br>7.258±1.092
	Monocytes (%)	Male<br>Female	7.904±0.990<br>7.540±1.273	8.080±1.190<br>9.420±2.621	9.034±2.280<br>9.644±2.240

	IgE^+ cells on lymphocytes (%)	Male<br>Female	1.216±0.906<br>1.338±0.523	2.142±1.148<br>1.044±0.677	2.064±1.583<br>0.994±0.558
	NK cells	Male<br>Female	3.990±0.564<br>3.580±0.724	3.986±0.734<br>4.742±1.359	3.542±1.277<br>7.102±2.662*[1]
	Activated NK cells (%)	Male<br>Female	48.20±6.119<br>28.34±5.126	45.72±6.433<br>25.86±3.325	42.38±4.464<br>25.66±3.122
Spleen FACs	Lymphocytes (%)	Male<br>Female	58.86±5.590<br>57.18±4.124	57.40±4.094<br>56.00±6.665	62.12±5.999<br>54.54±4.787
	T-cells (%)	Male<br>Female	27.94±3.431<br>27.80±3.050	29.08±3.163<br>27.50±6.131	27.94±4.448<br>25.06±6.135
	$CD4^+$ T-cells (%)	Male<br>Female	53.50±2.540<br>53.40±1.459	53.92±3.330<br>52.40±4.743	52.70±0.704<br>53.90±5.178
	Activated $CD4^+$ T-cells (%)	Male<br>Female	45.06±6.408<br>46.02±6.423	35.88±5.585<br>42.58±11.24	35.78±7.917<br>50.80±4.904
	$CD8^+$ T-cells (%)	Male<br>Female	27.78±1.293<br>26.84±0.844	27.58±1.970<br>26.72±5.969	28.42±0.729<br>24.76±4.438
	Activated $CD8^+$ T-cells (%)	Male<br>Female	25.18±5.134<br>22.10±3.395	23.52±4.061<br>22.64±8.740	24.64±7.823<br>27.08±8.113
	B-cells (%)	Male<br>Female	58.64±2.071<br>58.98±3.650	57.96±1.783<br>59.40±3.305	60.12±3.805<br>61.28±6.465
	Immature B-cells (%)	Male<br>Female	82.06±1.009<br>80.34±2.648	80.58±2.974<br>77.74±5.652	80.72±1.254<br>78.40±4.128
	Mature B-cells (%)	Male<br>Female	8.540±0.152<br>10.40±2.723	9.940±1.922<br>13.12±3.916	10.00±1.578<br>11.74±3.440
	Double negative B-cells (%)	Male<br>Female	8.860±0.924<br>8.860±0.564	8.860±1.106<br>8.560±1.826	8.760±1.324<br>9.360±0.910
	Monocytes (%)	Male<br>Female	5.080±0.460<br>5.880±0.630	4.620±0.356<br>5.620±0.522	5.140±0.577<br>5.540±1.014
	IgE^+ cells on monocytes (%)	Male<br>Female	1.000±0.418<br>0.480±0.455	1.360±0.513<br>0.520±0.466	0.760±0.467<br>0.620±0.130
	NK cells (%)	Male<br>Female	2.460±0.546<br>2.140±0.321	3.100±1.298<br>3.180±1.262	2.100±0.235<br>3.200±1.173
	Activated NK cells (%)	Male<br>Female	49.44±3.679<br>39.54±5.984	45.88±4.492<br>31.46±1.576	46.02±6.464<br>35.40±3.583

*The relative abundance of NK cells (%) in female *Tspo*$^{-/-}$ mice were significantly greater than in female *Tspo*$^{+/+}$ and in female *Tspo*$^{+/-}$ mice ($P<0.05$; ANOVA). Values represent mean ± standard deviation (n=5 per group).

Supplementary Table 4. PCR Primers

Genotyping primers			
**Name**	**Sequence (5'-3')**		
P1	GGTAGACTAGTGTGGGAAGATTTGA		
P2	ATGGTGATTGCAACTGATGTTC		
P3	TAGATACTGACCCTATCTGGGATGT		
**RT-qPCR primers**			
**Gene**	**Forward primer (5'-3')**	**Reverse primer (5'-3')**	**Reference**
Actb	GGACCTGACGGACTACCTCATG	TCTTTGATGTCACGCACGATTT	1
P450scc	ACATGGCCAAGATGGTACAGTTG	ACGAAGCACCAGGTCATTCAC	2
Gapdh	CCATGGAGAAGGCTGGGG	CAAAGTTGTCATGGATGACC	3
StAR	TCTCTAGTGTCTCCCACTGCATAGC	TTAGCATCCCCTGTTCGTAGCT	2
Tspo	GGGAGCCTACTTTGTGCGTGG	CAGGTAAGGATACAGCAAGCGGG	4
Tspo2	CCAGTCGGTGTGAGGATGAG	AGTAGAGACCAAGGGGCAGT	
**RT-PCR primers**			
**Gene**	**Forward primer (5'-3')**	**Reverse primer (5'-3')**	
Tspo	AACCAGCGACTGCGTGAG	GTGGCTTCCTGGGCTAAGAC	

Supplementary References

1 Morita, H. *et al.* Neonatal lethality of LGR5 null mice is associated with ankyloglossia and gastrointestinal distension. *Mol Cell Biol* **24**, 9736-9743 (2004).

2 Bouma, G. J., Hart, G. T., Washburn, L. L., Recknagel, A. K. & Eicher, E. M. Using real time RT-PCR analysis to determine multiple gene expression patterns during XX and XY mouse fetal gonad development. *Gene Expr Patterns* **5**, 141-149 (2004).

3 Dveksler, G. S., Basile, A. A. & Dieffenbach, C. W. Analysis of gene expression: use of oligonucleotide primers for glyceraldehyde-3-phosphate dehydrogenase. *PCR Methods Appl* **1**, 283-285 (1992).

4 Kam, W. W., Meikle, S. R., Dunstan, C. R. & Banati, R. B. The 18 kDa translocator protein (peripheral benzodiazepine receptor) expression in the bone of normal, osteoprotegerin or low calcium diet treated mice. *PloS one* **7**, e30623 (2012).
